# Pathogenicity and virulence of *Yersinia*

**DOI:** 10.1080/21505594.2024.2316439

**Published:** 2024-02-22

**Authors:** Jarett A. Seabaugh, Deborah M. Anderson

**Affiliations:** Department of Veterinary Pathobiology, University of Missouri, Columbia, USA

**Keywords:** Yersinia, pestis, pseudotuberculosis, enterocolitica, plague, yersiniosis

## Abstract

The genus *Yersinia* includes human, animal, insect, and plant pathogens as well as many symbionts and harmless bacteria. Within this genus are *Yersinia enterocolitica* and the *Yersinia pseudotuberculosis* complex, with four human pathogenic species that are highly related at the genomic level including the causative agent of plague, *Yersinia pestis*. Extensive laboratory, field work, and clinical research have been conducted to understand the underlying pathogenesis and zoonotic transmission of these pathogens. There are presently more than 500 whole genome sequences from which an evolutionary footprint can be developed that details shared and unique virulence properties. Whereas the virulence of *Y. pestis* now seems in apparent homoeostasis within its flea transmission cycle, substantial evolutionary changes that affect transmission and disease severity continue to ndergo apparent selective pressure within the other *Yersiniae* that cause intestinal diseases. In this review, we will summarize the present understanding of the virulence and pathogenesis of *Yersinia*, highlighting shared mechanisms of virulence and the differences that determine the infection niche and disease severity.

## Introduction

Current taxonomy places the Yersiniacea family within the order Enterobacterales, distinct from the previous classification with Enterobacteriaceae. Within this Yersiniacea family are pathogens that infect humans, animals, plants, and insects. The *Yersinia* genus includes 26 classified species, some of which cause significant human diseases including pandemic bubonic and pneumonic plague. Although the pathogenic species of *Yersinia* share many genetic similarities, numerous differences define their unique life cycles and virulence properties. With the ease of genomic technology worldwide, there are presently well over 500 whole genome sequences of isolates of the pathogenic *Yersiniae*, approximately 80–90% of which are *Y. pestis* [1]. Worldwide collections of ancient DNA from the remains of hundreds of humans have resulted not only in the positive confirmation of *Y. pestis* as the causative agent of the black plague, but also from the collective database and sequence analysis, the evolutionary path that led to flea transmission and historically explosive outbreaks of plague can be traced [2,3].

Within the *Yersinia* genus, *Y. enterocolitica* is evolutionarily distinct from the *Y. pseudotuberculosis* complex, which includes four pathogens that have a high degree of similarity at the DNA level: *Y. pseudotuberculosis*, *Y. similis*, *Y. wautersii*, and *Y. pestis*. *Yersinia pestis* is the most divergent member of this cluster, having acquired enhanced virulence and flea transmission around 5,000 years ago [4,5]. Since its initial emergence, *Y. pestis* has evolved into biovars which are stably maintained in the environment in the flea-rodent transmission cycle, and mutations that weaken this cycle are predicted to be unable to persist. More than 300 phenotypic changes between *Y. pestis* and its nearest *Y. pseudotuberculosis* relative have occurred that led to enhanced invasive virulence and flea transmission of *Y. pestis*. Despite these differences, *Y. pestis* is highly similar at the DNA level to *Y. pseudotuberculosis*. The sequence divergence of both species from *Y. enterocolitica*, however, is substantial [6,7]. In fact, the divergent evolution of *Y. enterocolitica* characterizes its ongoing acquisition of enhanced virulence as an enteric pathogen. Recent studies indicate further classification of *Y. enterocolitica* at the subspecies level.

### Pathogenesis and transmission of plague

*Yersinia pestis* is the causative agent of the plague, which comes in three forms depending on the route of transmission as well as the progression of infection. Modern plague most often begins with the bubonic form following transmission by flea bite [8]. Pulmonary or direct intravenous inoculation of *Y. pestis* leads to the deadliest forms of the disease wherein only days separate infection and mortality, with a short window available for successful antibiotic treatment [9]. Flea transmission of *Y. pestis* depends on the *Yersinia* murine toxin (Ymt), a bacterial lipase that is encoded on a plasmid associated with the evolution of flea transmission [10–13]. Ymt plays a critical role in the early colonization of the flea, acting during bloodmeal digestion to protect from bacteriocidal mechanisms and contributes to the formation of a bacterial cast that facilitates transmission [11,14,15]. In addition to Ymt expression, *Yersinia* also produce biofilm in the flea midgut which enhances transmission during subsequent feeding [16]. Four proteins encoded by the haem storage operon (*hms*) are involved in production of a secreted exopolysaccharide (EPS) matrix that forms the basis of the biofilm when *Y. pestis* colonize the flea midgut [17,18]. Without *hms* expression, *Y. pestis* are fully virulent in the mouse or rat model of bubonic plague when inoculated by needle, indicating their essential functions are confined to fleas [19]. Whereas *hms* is present in all circulating biovars of *Y. pestis*, it is not uniformly found in clinical strains of *Y. enterocolitica* and *Y. pseudotuberculosis* [20]. These observations suggest that colonization of the insect intestinal tract requires distinct functions compared to the mammalian gut.

The evolution of flea transmission by *Y. pestis* compared to its most recent ancestral species, *Y. pseudotuberculosis*, has been tracked to a small number of horizontal acquisition events that enabled effective digestion, adherence, and growth in the flea midgut [21]. Although *Y. pseudotuberculosis* is able to synthesize the EPS biofilm, infection is toxic to fleas due to the production of a surface urease enzyme able to degrade urea into ammonia [22]. The inactivation of urease in *Y. pestis* is due to a frameshift mutation in *ureD* that introduces a stop codon thereby abrogating the potential for toxicity in fleas. This loss of function mutation combined with three other loss of function events that resulted in increased biofilm formation, as well as Ymt, are sufficient to confer the stable colonization of *Y. pseudotuberculosis* in the flea midgut [23].

### Pathogenesis and transmission of yersiniosis

Both *Y. enterocolitica* and *Y. pseudotuberculosis* cause yersiniosis, an acute gastroenteritis in humans and agricultural animals, especially swine [24]. Each of these pathogens is resistant to cold temperatures and grows well in refrigerated food or blood, which has been associated with an increased probability of direct or foodborne transmission [25]. Yersiniosis is endemic in Europe and South America, causing periodic outbreaks, with *Y. enterocolitica* being responsible for the vast majority of human and animal cases. Nevertheless, the disease occurs worldwide, with human cases also reported in Africa, Asia and North America [26–28]. Although the disease is often self-limiting, severe manifestations are not uncommon and can progress to mesenteric lymphadenitis, reactive arthritis, and sepsis. There are at least six biotypes of *Y. enterocolitica*, and within these are specific O-antigen serotypes [29–31]. Significant genetic differences have been identified between biotypes that define an evolutionary path for *Y. enterocolitica* that, like its invasive *Y. pestis* relative, have resulted in evolved strains with enhanced capability for virulence and transmission. For example, biotype 1A strains do not carry the plasmid-encoded type III secretion system (T3SS) and lack adherence factors as well. Though once thought to be of low virulence, the type 1A strains that carry an alternative enterotoxin are commonly isolated in the environment and from animal or human samples [32]. In contrast, the remaining biotypes (1B, 2, 3, 4, and 5) carry the T3SS plasmid and accessory factors that promote adherence, such as the attachment and invasion locus (*ail*), or that encode iron-binding siderophores. Biotypes 1B, 3, and 4 are the most prevalent, and in addition to the T3SS and Ail, these three biotypes express the horizontally acquired heat-stable enterotoxin which induces gastrointestinal epithelial cells to cause hyper-secretion diarrhoea in infected animals and humans [33]. More than 80% of human infections are caused by biotypes 3 and 4, mostly of the O:3 serotype [29,31]. The O-antigen is thought to contribute directly to the virulence of *Y. enterocolitica*, allowing for enhanced resistance to anti-microbial peptides and other hydrophobic anti-microbial agents as well as complement (see below) [34–36]. *Y. entercolitica* biotypes can be geographically restricted. For example, in Europe, the most commonly reported biotype is 4, whereas in China, biotype 3 is most common [37,38]. Other serotypes, for example biotype 1A and 2, can be found worldwide in high prevalence [39].

### Evolutionary divergence of pathogenesis: Yersinia adhesins

#### Acquisition of adhesins by *Yersinia pestis* facilitated flea transmission

One of the key features of *Yersinia* is its ability to adhere to mucosal tissue [40]. Recognition of host cell surface receptors as well as evasion of mucosal immunity are mediated by a handful of extracellular proteins produced by pathogenic *Yersinia* species. Expression of surface proteins that enhance adherence and invasion is an area of divergence between *Y. pestis* and the other pathogenic *Yersiniae*, and not unexpectedly, this diversion is due to the sum of both the acquisition and loss of genetic information. For *Y. pestis*, pathogenesis in the mammalian host begins at the periphery, with adherence and invasion linked to a recently acquired broadly acting secreted protease, plasminogen activator protease (Pla), found in the modern lineage of *Y. pestis*. Pla is a β-barrel outer membrane aspartate protease that is exported across the inner membrane due to its NH_2_-terminal signal peptide [41]. Autoproteolysis of surface localized Pla activates its functions against numerous host and bacterial targets including key mammalian defence proteins in the fibrinogen family [42]. Plasminogen cleavage results in active plasmin which acts as a broad-acting serine protease that breaks up fibrin clots. The breaking of these clots is thought to contribute to the pathogenesis of plague. While increased plasmin facilitates the dissemination of *Y. pestis*, it may also skew the inflammatory response [43,44].

Although these protease functions collectively mean that Pla is critical for invasion from the peripheral site when *Y. pestis* is transmitted by flea, it also plays a role in pulmonary disease, perhaps due to its role in adhesion [45–48]. Mutations that disrupt the protease activity of Pla do not abrogate its role in adhesion, suggesting bifunctional activities [49]. In addition to *pla*, the pPCP1 plasmid also encodes for a secreted bacterial toxin, pesticin (Pst), that also distinguishes the capabilities of *Y. pestis* from *Y. pseudotuberculosis* [41]. Pesticin is a muramidase that is toxic to bacteria that carry the chromosomal high-pathogenicity island (HPI) encoding for the synthesis and transport of the siderophore yersiniabactin (Ybt). The pesticin receptor (Psn) is an outer membrane protein that binds and transports Ybt-Fe and is encoded in the HPI. This genetic locus is found in *Y. pestis* as well as related gram-negative pathogens including *Y. pseudotuberculosis* and some strains of *Y. enterocolitica*, *E. coli*, and *K. pneumoniae* [50,51]. Following binding to Psn, the muramidase activity digests the peptidoglycan of the target cell, creating spheroplasts that readily lyse under environmental stress [52–54]. Thus, without the co-expression of an immunity protein found in *Y. pestis* that can inactivate Pst, the expression of Psn makes the bacterial cell sensitive to Pst-induced lysis. Whole genome sequencing and multiple-locus variable-number tandem repeat analysis (MLVA) indicate the existence of virulent *Y. pestis* strains without pPCP1 as well as those with mutated versions of Pla, all of which carried significant pathogenic potential for causing plague in at least some vertebrate hosts [1,55–57].

Bioinformatic and genetic evidence indicate that *Y. pestis* utilizes multiple mechanisms for adhesion to epithelial cells that may play overlapping roles during infection. For example, *Y. pestis* adherence to respiratory epithelial cells can be mediated by at least three adhesins: Pla, pH6 antigen (Psa), and capsular protein F1 (CaF1), with each playing an apparent redundant function in promoting adherence to host cells [45]. Encoded by the pMT1 plasmid and therefore unique to *Y. pestis*, F1 is an extracellular polymer that forms small, hairlike pili that cover the bacterial surface [58,59]. Expression of F1 is also thought to contribute to immune evasion, providing protection from phagocytosis [60]. Nevertheless, the polyprotein is an immunodominant antigen used to document the seroconversion of animals and humans to *Y. pestis*. Furthermore, antibodies to F1 are protective in experimental rodent models of plague, yet its function during mammalian infection appears dispensable to pathogenesis [61,62].

**Figure 1.**
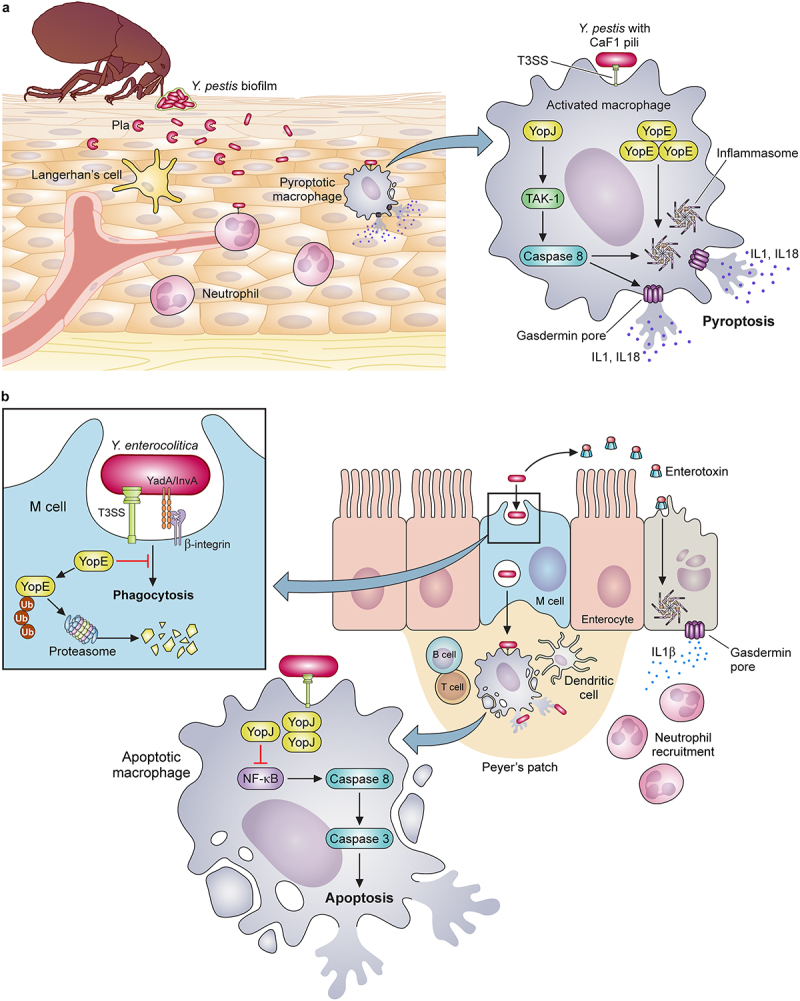
*Evolutionary changes impacting adherence and invasion characterize enhancements to the virulence potential of pathogenic Yersiniae*. (a) Model depicting the interactions unique to the flea transmission cycle of *Y. pestis*, including secretion of the Pla protease that promotes the invasion of the dermal tissue. Early host-pathogen interactions preferentially occur with phagocytic cells (mainly macrophages and neutrophils) where the T3SS-mediated injection of YopE and YopJ, along with the other Yop effectors not shown, activate two parallel mechanisms for rapid pyroptosis: 1) YopJ inhibition of signaling through host TAK-1 allows caspase 8-activation of the inflammasome; 2) abundant YopE in the cytoplasm activates the pyrin inflammasome. (b) Model depicting the interactions involving *Y. enterocolitica* and the intestinal mucosa due to: 1) the abundant expression of adhesins YadA and InvA; 2) secretion of heat-stable toxin (ST), and 3) injection of Yops. Intoxication of the gut epithelium by ST causes disruption of cilia and pyroptosis, which initiates neutrophil recruitment. Adherence to M cells and other IECs is facilitated by the clustering of host β-1 integrin, which initiates phagocytosis of *Yersinia*. Injection of YopE has little anti-phagocytic impact due to proteasome degradation. Bacteria transit through M cells allowing infection of the Peyer’s patches (grey). Within Peyer’s patches, *Yersinia* targets tissue-resident macrophages, where YopE is anti-phagocytic and abundant YopJ injection induces apoptosis and anti-inflammatory responses. Created with BioRender.com.

Attachment Invasion Locus (Ail) protein can be found on the cell surface of any of the pathogenic *Yersinia* species where it is known to mediate host cell attachment, invasion, and resistance to mammalian complement [63]. Abundantly expressed at ambient and mammalian temperatures in *Y. pestis*, Ail is thought to be more weakly expressed by *Y. pseudotuberculosis* and *Y. enterocolitica* [64]. Ail expression enhances resistance to complement by acting on factor H and the C4 binding protein, thereby preventing assembly of the complement complex on *Yersinia* [63,65–67]. Ail has also been shown to play an essential role in the T3SS through an interaction involving its fibronectin-binding (FNB) domain [68]. This interaction appears essential for the insertion of the T3SS needle, suggesting that in addition to its role in promoting adherence to cells, Ail may contribute to host cell specificity for targeting by the T3SS (see below). Genetic data indicate the importance of Ail in mediating invasion as a consequence of binding via its FNB domain, such that the mutations that increased the strength in fibronectin binding correlate with enhanced invasive properties of *Y. pestis* [69]. Overall, the data indicate an essential role for Ail in *Y. pestis* pathogenesis (Figure 1a).

## Binding to host cell integrins promotes invasion of intestinal epithelium by enteric Yersiniae

Yersinia Adhesion A (YadA) is secreted by a T5SS mechanism but is only expressed by *Y. pseudotuberculosis* and *Y. enterocolitica*. *Yersinia pestis* fails to express YadA due to a silencing frameshift mutation [70]. YadA is encoded on pYV, the T3SS plasmid of enteropathogenic *Yersiniae*, and its expression is coordinated by the low calcium response [65]. YadA-mediated adhesion plays a critical role in the pathogenesis of *Y. enterocolitica* but was dispensable for oral infection of mice by *Y. pseudotuberculosis*. For the enteropathogenic *Yersiniae*, YadA can also mediate resistance to complement and may be functionally redundant to Ail for this immune evasive mechanism [71]. Like Ail, YadA contains an FNB domain and is thought to bind to complement factor H and C4 binding protein [72,73]. Unexpectedly, however, restoration of *yadA* expression to *Y. pestis* caused attenuation of virulence in the mouse model, indicating its function is not redundant to Ail during plague [74]. This observation suggests that the unique adherence properties of YadA attenuate the virulence of *Y. pestis* by altering host cell specificity for infection. YadA is known to bind beta-1 integrins on the surface of epithelial cells and other cells that line the intestinal tract via its FNB domain. This interaction facilitates the invasion of epithelial cells and M cells in the intestinal tract, required for the colonization of Peyer’s patches (Figure 1b).

Invasin (InvA) is also a cell surface protein secreted by a type V mechanism with FNB domains that mediate binding integrins on the plasma membranes of mucosal cells [75]. For *Y. pseudotuberculosis* and *Y. enterocolitica*, InvA is strongly expressed at environmental temperature (26℃) and more weakly expressed at 37℃. However, low pH may induce higher levers of InvA expression at 37℃ [76]. Temperature-dependent regulation of InvA is also associated with specific serotypes [77]. The amount of surface-exposed InvA is relevant to the outcome of adherence to the host cell. For example, high surface-localized InvA promotes clustering of host integrins, thereby leading to the internalization of *Yersinia* by M cells in the intestine [78]. In contrast, low levels of InvA stimulate signalling pathways that activate inflammatory responses. Like YadA, however, InvA is not produced by *Y. pestis*, leading to the hypothesis that these two adhesins play critical roles in determining an enteropathogenic infectious cycle [66]. Indeed, for *Y. enterocolitica*, InvA and YadA were required for invasion of Peyer’s Patches via M cells in the mouse gastrointestinal tract (Figure 1b) [79–81]. This process was necessary for vascular dissemination. Overall, differences in mechanisms of adherence and invasion are likely critical determinants of the early infection that dictate the unique replicative niche of *Y. pestis* compared to the enteric *Yersiniae*.

### Yersinia type III secretion system

In addition to all the functions described, adhesins are important to the functioning of the type III secretion system (T3SS) as well, presumably by facilitating intimate adherence with cells for insertion of the T3SS translocation pore [66]. The T3SS plays a dominant role in immune evasion for all pathogenic *Yersinia*, with a high degree of amino acid similarity between the machinery and effector proteins of each species. The T3SS is a transport and delivery device that injects effector Yersinia outer proteins (Yops) into host cells to prevent phagocytosis, block inflammatory cytokine production, and modify signalling pathways involved in programmed cell death [82,83]. The time, energy, and nutrient costs required for functional type three secretion causes negative feedback on *Yersinia* growth, a process known as the low calcium response (LCR) [84]. In laboratory media lacking calcium, *Yersinia* incubation at 37℃ results in high-level activation of the T3SS which causes severe growth restriction, whereas the addition of calcium to the media blocks secretion and allows growth at 37℃ [85,86]. Likewise, mutation of the secretion machinery results in loss of the low calcium response, suggesting bacteria sense secretion competence, or even target cell contact, and this causes feedback on metabolism and growth [87,88]. Early studies identified multiple genes that could be mutated and affect the LCR without having adverse effects on secretion [89]. These were later shown to be caused by negative regulators that sense or respond to environmental cues to activate secretion. In fact, the T3SS of *Yersinia* is tightly controlled at the transcriptional and post-transcriptional levels, allowing precise fine-tuning and balance between T3S and bacterial growth during infection.

## T3SS expression control

The LCR and all of the structural and effector proteins are encoded on the respective virulence plasmid for each *Yersinia* species (pCD1 for *Y. pestis*, pYV in the enteropathogenic strains) [89,90]. To ensure proper timing of expression, *Yersinia* utilizes a two-fold control mechanism for T3SS protein expression: temperature and cell-cell contact [85,91]. At lower temperatures plasmid-encoded Yersinia Modulator A (YmoA) prevents transcription of *lcrF*, an AraC-like DNA binding protein that activates the transcription of all T3SS genes [92–96]. When pathogenic *Yersiniae* enter the mammalian host, the shift in temperature from 25°C to 37°C causes a cascade of changes in the bacterium concerning many virulence factors, including the T3SS. Thermal instability of YmoA allows its degradation by the Lon and ClpXP proteases, releasing its block on *lcrF* transcription [97]. Additionally, the *lcrF* mRNA transcript itself includes a long untranslated leader sequence that forms tertiary structures at lower temperatures that bury the ribosome binding site [93]. With the increase in temperature, not only is YmoA degraded but this stem-loop structure is destabilized, thereby exposing the translation initiation sequences that allow for the production of LcrF protein. A third layer of temperature regulation that is thought to contribute to control over T3SS protein expression is changes in DNA supercoiling at 37°C [98]. These combined mechanisms allow for a tightly controlled, thermally responsive expression of the T3SS.

**Figure 2.**
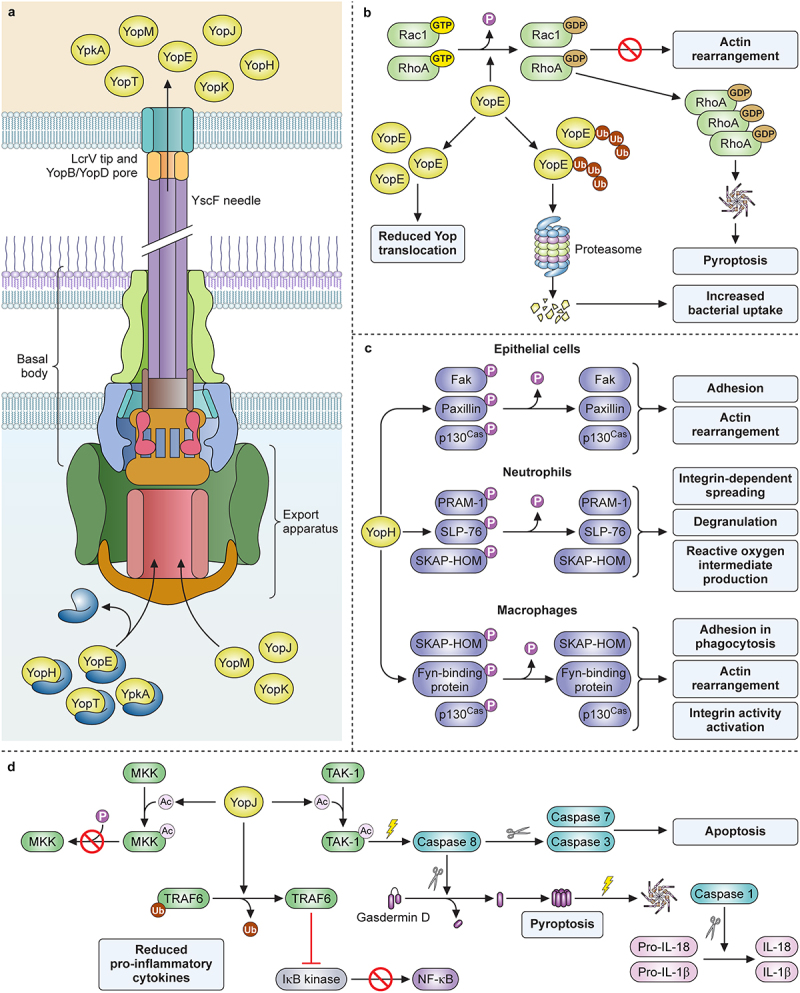
*Yersinia T3SS structure and contributions to pathogenesis by YopE, YoH, and YopJ*. (a) chaperones keep YopE, YopH, YpkA, and YopT in an unfolded, secretion-ready state as they are transported to the export apparatus. YopJ, YopM, and YopK do not require a chaperone for secretion. Following chaperone dissociation, Yops are transported through the sorting platform, basal body, needle, and translocon, then enter directly into the host cytoplasm. (b) Ubiquitination of YopE affects its function, as deubiquitinated YopE is associated with reduced Yop translocation while ubiquitinated YopE is degraded by host proteosomes and is associated with increased bacterial uptake. Functionally, YopE activates the GTPase domains of Rac1 and RhoA, causing them to be converted to their GDP-bound inactive forms. This inactivation prevents actin rearrangement, but the buildup of inactive RhoA can cause activation of the inflammasome and subsequent pyroptosis. (c) YopH dephosphorylates select targets in epithelial cells, neutrophils, and macrophages, blocking distinct functions in each. (d) YopJ acetylates MKK, preventing its downstream phosphorylation and activation. It also deubiquitinates TRAF6, preventing its downstream activation of IκB kinase and NFκB production. Lastly, YopJ acetylates TAK1, inactivating it and causing subsequent activation of caspase 8. Active caspase 8 can then cleave gasdermin D or cleave and activate caspase 7 and caspase 3. Cleavage and activation of caspase 7 and caspase 3 promotes apoptosis, while cleavage of gasdermin D causes it to form a membrane pore. This event signals the activation of the inflammasome and caspase 1 activation with subsequent production and release of IL-18 and IL-1β. Created with Biorender.com.

Expression and assembly of the T3SS machinery occur in response to the thermal cue as an ordered process that is understood at the genetic and biochemical levels (reviewed in [99]. The T3SS machinery forms a large protein complex that spans the inner and outer membranes of *Yersinia* (Figure 2a). Upon completion of the basal body and export apparatus, secretion of YscF allows the polymerization of a hollow needle on the surface that is capped by the protective antigen LcrV [100]. Contact between the needle and host cells provides a second activation signal that directly mediates transcriptional and post-transcriptional activation of T3S [101].

The response to such cues for the ordered expression and assembly of the T3SS is critical to pathogenesis as maximal T3SS activity comes with a negative impact on bacterial growth. It is also critical that effector proteins act rapidly after host cell contact in order to evade the phagocytic response. To achieve this balance, there must be communication at each stage. For example, two prominent proteins involved in substrate secretion regulation are YscP and an inner membrane protein YscU. YscP forms a polymer anchored in the inner membrane ring and acts as a molecular ruler within the YscF polymer needle as it forms. When the YscP polymer senses the appropriate needle length, it signals a switch from the secretion of machinery components to the secretion of the translocator apparatus components LcrV, YopD, and YopB [102]. Reduced expression of YscP results in a shorter needle, whereas over-expression results in longer polymers and longer needles. For *Yersinia enterocolitica*, YscP has been shown to contain separate domains for its secretion and for controlling substrate specificity [103,104]. The inner membrane protein YscU mediates hierarchy and responds to the readiness of the injection apparatus. Autocleavage at the carboxy terminus of YscU acts as a substrate specificity switch from middle substrates to effector substrates that will be injected into the host cell [105].

The translocator protein YopD also functions in the bacterial cytoplasm as a link between the LCR and the insertion of the pore in the host plasma membrane. Within the bacterial cell, YopD complexes with a chaperone LcrH and regulates expression of Yops in the presence of Ca^2+^ through binding to the untranslated leader of the mRNA [106,107]. This binding occludes the ribosome binding site and destabilizes the mRNA [108]. Through such targeting, effector Yop translation is negatively regulated in the presence of cytoplasmic YopD. Following secretion of YopD for assembly of the translocation pore, the mRNA is stabilized and the resulting translated Yop effectors can be secreted rapidly, perhaps even co-translationally, meaning as they are synthesized they can be initiated into the secretion pore [109,110]. LcrQ is another negative regulator of Yops which, like YopD, is a secretion substrate that facilitates the LCR while in the bacterial cytoplasm [111–113]. Secretion of LcrQ is thought to be a very early event following host cell contact that depletes the bacterial cytoplasm of this regulator, leading to rapid amplification of gene expression [114]. Over-expression of LcrQ causes decreased expression of LcrF likely through the binding of LcrQ to the ribosome to suppress translation. This consequently shuts down the T3SS [115]. These observations lead to a model whereby LcrQ secretion acts as a cue for host cell contact and translocation capability.

## T3SS translocation pore

The T3SS needle is comprised of multiple 6kDa YscF subunits and reaches 60-80 nm from the cell with an inner diameter of about 2 nm [116]. Once the needle length is established, LcrV is secreted, followed by translocator pore proteins YopB and YopD after host cell contact [117]. LcrV forms a pentamer at the tip of the YscF needle and is surface exposed where antibodies can bind and neutralize translocation [100,118]. Expression of the *lcrGVHyopBD* operon allows for co-regulation of the proteins necessary for forming the translocation pore [119]. The secretion of LcrV and subsequent formation of the needle tip signifies the formation of the injection-ready T3SS, with cell-cell contact between *Yersinia* and host cells acting to trigger the insertion of the pore [120]. *Yersinia* mutants lacking LcrV, YopD, and/or YopB are avirulent due to their inability to inject Yops into host cells. While injection of Yops by these mutants is abrogated, Yop secretion into the extracellular space by the Ysc machinery is not impacted [121–123].

Specificity for the T3SS injection of phagocytic cells is mediated by LcrV binding to *N*-formylpeptide receptor (FPR1) [124]. FPR1 is a G-protein coupled receptor expressed on the surface of myeloid cells including macrophages, neutrophils, and dendritic cells [125]. During *Y. pestis* infection, the interaction between FPR1 and LcrV is a critical virulence determinant, as loss of this interaction yields avirulent *Y. pestis*. However, mice lacking FPR1 appeared moderately more resistant to infection, indicating additional critical functions for LcrV. LcrV is highly conserved between *Yersinia* species, though anti-LcrV antibodies are not always cross-protective [126]. Hyper-variable regions exist that may allow for the fine-tuning of target cell specificity between pathogenic lifestyles of the different *Yersiniae*.

In addition to their role in the translocation of Yops, YopD and LcrV may have additional roles in modifying the host response to infection as both YopD and LcrV have been found to localize to the host cell cytoplasm [127,128]. Furthermore, functional homologs of YopB, YopD, and LcrV are found in most T3SSs expressed by gram-negative bacteria, where strong evidence supports their direct roles on the host cell response [129]. Insertion of the translocation pore into host membranes connects the cytoplasm of the two cells, allowing direct injection of effector Yops into the host cytoplasm. However, there is also host detection of the needle and pore that can activate the production of inflammatory cytokines, an outcome that is overcome by the action of the injected effector proteins [130].

## Yops

The effector and translocator Yops have a high degree of sequence similarity at the DNA and amino acid level between *Yersinia spp*., and consequently, they are thought to have similar functions for each pathogen [131]. In addition to translocator Yops B and D, there are at least seven effector proteins injected into the cytoplasm utilized by *Yersinia* for the disruption of the host response: YopJ (YopP in *Y. enterocolitica*), YopM, YopE, YopT, YopH, YpkA (YopO in *Y. enterocolitica*), and YopK (YopQ in *Y. enterocolitica*) (Figure 2a) [132]. Listed below are summaries of the anti-host roles played by the membrane and cytoplasmic Yops during *Yersinia* pathogenesis.

### YopB

With two transmembrane domains, YopB is the main translocation pore component [133,134]. Purified YopB is capable of disrupting cell membranes, much like its homologs IpaB of *Shigella flexneri* and PopB of *Pseudomonas aeruginosa* [135,136]. “Calcium-blind” *Yersinia* mutants that constitutively secrete Yops will secrete YopB into the extracellular milieu, suggesting it is in fact secreted prior to cell contact and makes up a portion of the needle tip complex [137,138]. This hypothesis is supported by similar observations in other gram-negative T3SSs but has yet to be demonstrated in *Yersinia* [139].

### YopD

Containing only one transmembrane domain, YopD and its homologs are commonly called the minor translocators [134]. Like YopB, *Yersinia’s* successful injection of Yops for disarming the host immune response is dependent on properly functioning YopD. YopB and YopD are known to interact in an oligomeric membrane complex [123]. In addition, YopD is exposed to the host cell cytoplasm where it may function to regulate effector Yop translocation through its interaction with YopK, though the overall topography of YopD has not yet been described and the host proteins that may also be involved in regulating the translocation pore have not been identified [138]. Extensive mutagenesis on YopD has been conducted, revealing distinct amino acids required for specific functions in the translocation and regulation of Yop effectors. However, an overall understanding of the biochemical properties of YopD is lacking.

### YopE

The activity of YopE as a molecular mimic that acts on mammalian Rho GTPases has been known for some time, and it is clear that all pathogenic strains of *Yersinia* heavily depend on YopE during their pathogenic cycles [82,140,141]. In the host, Rho GTPases are master regulators of cytoskeleton function, particularly cytoskeletal rearrangements involving bundling or filamentation of actin [142]. YopE is a GTPase activating protein (GAP) that targets G-proteins tethered to the plasma membrane [143,144]. YopE activates the GTPase activity of RhoA and Rac1, causing them to shift from their GTP-bound active state to a GDP-bound inactive state, effectively preventing cytoskeletal rearrangements necessary for phagocytosis (Figure 2b) [145,146]. However, cytoplasmic accumulation of inactive RhoA causes the host cell to activate pyrin inflammasome assembly, which leads to pyroptosis [147]. This outcome may be modulated by other Yop effectors discussed below, suggesting it likely has a deleterious impact on infection.

### YopH

Another inhibitor of phagocytosis, YopH is a highly active protein tyrosine phosphatase (PTP) that contributes essential functions to the pathogenesis of all pathogenic *Yersinia* [141,148,149]. There are likely many host targets that are normally phosphorylated and dephosphorylated for signal transduction, some of which are critical mediators of phagocytosis (Figure 2c) [150,151]. YopH activity can be visualized within only a minute of injection where it blocks actin rearrangement and the early steps of phagocytosis by host macrophages [152,153]. In macrophages, Crk-associated tyrosine kinase substrate (p130^Cas^), Fyn-binding protein, and SKAP-HOM are targeted, while in epithelial cells, focal adhesion kinase (FAK), p130^Cas^, and paxillin are the main targets [154,155]. In both cell types, the T3SS injection of YopH into host cells causes a potent effect on cytoskeletal rearrangement [156]. Perhaps one of its more important roles, however, is to inactivate neutrophils. The rapid action of YopH on inhibiting signal transduction through dephosphorylation of multiple target proteins, including PRAM-1 and SLP-76, inhibits neutrophil function and appears to strongly correlate with the ability of *Yersinia* to resist neutrophilic-clearance [157].

### YopJ

One of the most well-characterized effector Yops, YopJ (YopP in *Y. enterocolitica*) targets the MAPK and NFκB pathways of host cells [158,159]. Functionally, YopJ is classified as a cysteine protease due to the similarity of its active site to cysteine proteases of adenovirus. However, the primary biochemical activities of YopJ and its homologs in other T3SSs were later shown to be deubiquitinase and acetyltransferase, each requiring the same active site [160–162]. Loss of function mutation of the active site causes a null phenotype, indicating that these enzymatic activities are critical to the role of YopJ in *Yersinia* pathogenesis [159]. Deubiquitination of TRAF6 by YopJ prevents phosphorylation of IκB kinase, which would otherwise cause activation of NFκB and production of inflammatory cytokines as well as anti-apoptosis proteins (Figure 2d) [163]. As an acetyltransferase, YopJ has been demonstrated to acetylate the active site loops of proteins in the Map kinase kinase (MKK) family [164]. This acetylation prevents the phosphorylation of MKKs, effectively blocking one or more of the MAPK signalling cascades that contribute to the inflammatory response. Whereas some cell types respond to YopJ by activating caspase 8-dependent apoptosis, in activated macrophages, YopJ-dependent inhibition of TGF-β activating kinase (TAK-1) stimulates an alternative function for caspase 8 in its activation of caspase 1 and pyroptosis [165,166]. Thus, depending on the cell type that is injected by YopJ, the response may be apoptosis or pyroptosis, suppression or activation of pro-inflammatory cytokine production. Furthermore, *Yersinia* can modulate this outcome with the non-enzymatic effector Yops, YopM, and YopK (see below) [130,167].

### YopT

Along with YopE, YopT is an inactivator of Rho GTPases. Unlike YopE, however, which utilizes an arginine finger motif to target Rho GTPases with its GAP domain to convert them to their inactive form, YopT acts as a cysteine protease to cleave Rho GTPase from the host cell plasma membrane [168,169]. Also unlike YopE, which selectively targets Rho GTP in its active form and converts it to its GDP-bound inactive form, YopT shows no preference for GTP vs. GDP-bound conformation [168]. Both proteins preferentially target RhoA and Rac1, and both can cause pyrin inflammasome activation through this process [147,170]. While the functions of YopE and YopT may appear redundant, the phenotypes of deletion mutants are not identical, with the loss of *yopT* from *Y. pseudotuberculosis* having only negligible impact on virulence, whereas deletion of *yopE* severely attenuates virulence [171]. Likewise, during plague, YopT was also found to be dispensable in the mouse model [172].

### YpkA

YpkA also appears to be involved in the antiphagocytic response [173,174]. Like YopE and YopT, YpkA targets G-proteins RhoA and Rac1 [175]. YpkA is distinct, however, in that it contains two functional domains. The first is a serine/threonine kinase domain (Ser/Thr) while the second is a guanine nucleotide dissociation-like inhibitor (GDI) domain. The Ser/Thr domain of YpkA is found in the amino-terminal region of the protein while the GDI domain is found in the carboxy-terminal region, with these domains operating synergistically to inhibit phagocytosis [176]. The CH_3_-terminus of YpkA contains the actin-binding region and is responsible for YpkA activation only in the host cell cytoplasm. The Ser/Thr domain of YpkA phosphorylates GTP-binding protein Gαq, inactivating it, with this process being critical for its biological role in the host [132,177]. The inactivation of Gαq may have direct consequences on pathogenesis. Transgenic mice lacking Gαq suffer from defective platelet function which causes profuse bleeding even in the absence of infection [178]. While the Ser/Thr domain performs its phosphorylation function, the GDI domain interacts with RhoA and Rac1, locking them in their inactive GDP-bound forms. Together, these two functions of YpkA are thought to effectively block host cell cytoskeleton rearrangement, thereby protecting *Yersinia* from phagocytosis [177].

### YopM

YopM has no known enzymatic function, has no known homologs outside of *Yersinia*, and plays a key role in reducing the activation of the inflammasome [179]. Whereas loss of only YopM causes a relatively minor negative impact on virulence, deletion of both YopM and YopJ causes a synergistic toll on pathogenesis suggesting their coordinated action may be important [180,181]. YopM has been shown to bind host protein kinase C-related kinase 2 (PRK2) and ribosomal S6 protein kinase 1 (RSK1), acting as a scaffold that can disrupt the assembly of the pyrin inflammasome [182–184]. This is thought to be a significant role for YopM due to the activities of YopE, YopT, and YpkA that lead to cytoplasmic accumulation of inactive RhoA, which in normal instances would cause activation of the pyrin inflammasome and an inflammatory host cell death [147]. In addition to a role in the cytoplasm, YopM includes a nuclear localization signal and has been found in the nucleus of injected cells, where it may function indirectly to cause the production of anti-inflammatory cytokines [185]. Overall the mechanism of YopM in the nucleus is not understood, and the importance of PRK2/RSK1 binding to pathogenesis remains unclear [186]. For example, loss of *Y. pestis yopM* caused attenuation in the intradermal and intravenous mouse models of plague, though seemed less critical in the pneumonic model [187,188]. Similarly, intravenous injection of *Y. pseudotuberculosis yopM* resulted in decreased colonization of the spleen and increased neutrophil recruitment compared to the wild-type infection [189]. In contrast, the *Y. enterocolitica yopM* mutant remained fully capable of infecting mice through the oral route [148]. As discussed above, YopM may function as a modifier of the host response to YopE and YopJ activities, which may themselves have species-specific roles in pathogenesis. In support of this model, the phenotypic analysis of *Y. enterocolitica yopE* and *Y. pseudotuberculosis yopE* mutants mirror the phenotypes of the *yopM* mutant in the mouse models [141,190,191].

### YopK

As with YopM, there are no known homologs of YopK outside of *Yersinia*, yet its function is critical to the pathogenesis of yersiniosis and plague [140,190,192]. In vitro, loss of *yopK* (*yopQ* in *Y. enterocolitica*) causes a hyper-translocation phenotype wherein increased delivery of effector and translocator Yops occur which results in increased cytotoxicity in injected cells [138,193]. Early work also showed that the *yopK* mutant caused increased haemolysis of red blood cells due to the formation of an enlarged translocation pore in the plasma membrane [194]. Conversely, the over-expression of YopK resulted in a smaller translocation pore. The overall model became that the kinetics of delivery of translocator and effector Yops was controlled by the size of the translocation pore, with YopK playing an essential role in dictating the size. YopK can be co-precipitated with YopD and YopB and appears to localize to the translocation pore during T3SS injection [130]. However, this hypothesis has not been rigorously examined and genetic evidence for YopD involvement in pore size control is lacking. In addition, evidence for direct interactions between YopK and host cell proteins is also present, leaving the role of the YopD-YopK interaction of uncertain relevance to the underlying mechanism in pathogenesis.

A yeast two-hybrid screen selected for host proteins that could bind YopK, revealing the receptor for activated C kinase 1 (RACK1) as a potential candidate [195]. In mammalian cells, RACK1 is an abundant cytoplasmic protein present in most cell types that can bind to β1-integrin family members on the cytoplasmic face of the plasma membrane. Although RACK1 knockout mice have not been examined for susceptibility to *Yersinia* infection, epithelial cells expressing β1 integrin may represent a cell-specific function for YopK in pathogenesis. In addition, there is no reported role for RACK1 or β1-integrin in the modulation of membrane pores, leaving no clear connection between RACK1 and the regulation of Yop translocation. Perhaps the more important role for YopK in pathogenesis occurs in macrophages where it appears to modulate YopJ-dependent apoptosis in vitro [192]. Macrophages infected with the *yopK* mutant are more resistant to apoptosis, and this is thought to play an early and critical role in controlling inflammatory cytokine production in vivo.

## Evolutionary mechanisms controlling effector Yop activity provides niche-specific functions in pathogenesis

As the pathogenic *Yersinia* species have diverged, the activities of their respective Yops have undergone small evolutionary changes that impact their abundance and/or binding partners in host cells. In addition to its role in preventing phagocytosis, YopE may regulate effector translocation such that mutational inactivation of *yopE* causes a hyper-translocation phenotype [196]. Levels of YopE in the host cell cytoplasm may therefore be regulated by host cell proteins, which in turn, govern the kinetics of YopE function. For example, in M cells, YopE is rapidly ubiquitinated in the cytoplasm, leading to its degradation by the host proteasome thereby reducing its impact on phagocytosis [197]. Consequently, *Yersinia enterocolitica* infection of M cells may result in bacterial uptake which facilitates invasion of the Peyer’s patches. Mutation of the ubiquitination sites of *Y. enterocolitica* YopE also reduced translocation of other Yops, suggesting that YopE half-life may globally regulate Yop abundance in host cells [198]. Moreover, the loss of ubiquitin-regulated turnover of YopE caused attenuation of *Y. enterocolitica* in a mouse model of yersiniosis, indicating that regulated turnover of YopE is critical to pathogenesis.

Reduced, rather than increased, YopJ injection is an evolutionary step of *Y. pestis* associated with enhanced virulence. This was demonstrated through the creation and analysis of YopJ-YopP chimeras, where the secretion signals of YopJ (*Y. pestis*) and YopP (*Y. enterocolitica*) were swapped, resulting in the hyper-injection of YopJ by *Y. pestis*. Consequently, *Y. pestis*-killing of macrophages was enhanced, which attenuated, rather than benefitted, virulence in a murine plague model [199,200]. Further evidence supports the hypothesis that the concentration of YopJ in host cells influences the activation of TNF-dependent cell death such that low-level injection has a higher impact on TNF signalling [201]. The combined data indicate that control over YopJ abundance in the host cell may dictate cell-specific or pathogen-specific roles in pathogenesis.

In addition to its modified secretion signal, YopJ activity has evolved from the ancestral *Yersinia* lineage [202]. For example, leucine at codon 177 of *Y. enterocolitica* YopP is mutated to phenylalanine in some, but not all, *Y. pestis* as well as the *Y. pseudotuberculosis* strains. This substitution decreases the cytotoxic response that is stimulated by YopJ during infection of macrophages [203]. These observations indicate that YopJ-dependent cytotoxicity that is observed in vitro likely plays little role in the disease process. Consistent with this hypothesis, *yopJ* mutants show similar virulence properties as wild-type *Y. pestis*, *Y. pseudotuberculosis*, and *Y. enterocolitica* [140,148,173,181,191,204,205]. Nevertheless, functional YopJ/P is present in all *Yersinia* strains carrying the T3SS plasmid indicating strong evolutionary pressure for its retention.

### Pathogenic Yersinia control and manipulate the host innate immune response

#### Control over inflammasome-mediated host defense

Inflammasome-mediated host defence is a major component of innate immunity strongly active in phagocytic cells and has been the subject of intense research on *Yersinia* pathogenesis for the past 20 years. Upon assembly and activation of the inflammasome, caspase 1-mediated cleavage of cytokines and pore-forming proteins leads to a pyroptosis-type of host cell death and the release of mature cytokines IL1β and IL18 [206]. These cytokines allow for neutrophil recruitment and IFNγ production, both of which contribute to the clearance of *Yersinia* [207–209]. Initial studies indicated that insertion of the Yop secretion pore would stimulate the host NLRP3 inflammasome were it not regulated by translocated effector Yops [130]. YopJ, YopK, and YopM may work together to counter the activation of the inflammasome, thereby limiting the induction of pyroptosis in macrophages [180,210]. Further, mice lacking caspase 1 appear only modestly more sensitive to *Y. pestis* or *Y. pseudotuberculosis* infection consistent with its neutralization in vivo [211,212]. Collectively these observations indicate that the activation and subsequent inactivation of the inflammasome occur as a consequence of the T3SS.

## Immune suppressive outer membrane structure of *Yersinia*

Early host responses to would-be gram-negative bacterial pathogens involve recognition of pathogen-associated molecular patterns (PAMPs), including lipopolysaccharide (LPS), flagella, insertion of the T3SS pore, and internalization as well as invasion of host cell cytoplasm [210]. Host cells have numerous receptors for PAMPs, collectively referred to as pathogen pattern recognition receptors (PRRs). Membrane-bound receptors in the Toll family (Toll-like receptors, TLR) are PRRs that are commonly involved in stimulating innate immunity to bacterial infection and present a challenge for *Yersinia* as well as any would-be pathogen that must be overcome to avoid elimination in the mammalian host.

The *Yersinia* cell surface presents several immune evasive strategies that have been selected for by the unique aspects of the infectious niche of individual pathogenic strains. In many ways, the evolution of lipopolysaccharide (LPS) chemistry enhances the immune evasive properties of *Yersinia*. One of these is the O-antigen, the outermost domain of typical gram-negative LPS. Whereas *Y. pestis* has lost expression of O-antigen altogether due to frameshift mutations in 4 genes required for its biosynthesis and attachment, *Y. pseudotuberculosis* and *Y. enterocolitica* express these genes and full-length O-antigen [213]. For *Y. enterocolitica*, O-antigen synthesis has evolved with enhancements to virulence and is directly associated with complement evasion [39,214]. While *Y. enterocolitica* and *Y. pseudotuberculosis* are capable of surviving in the intestinal tract and causing GI symptoms, loss of O-antigen may have impacted the ability of *Y. pestis* to persist in the GI tract. In further support for the niche-specific importance of O-antigen, a limited number of serotypes of *Y. enterocolitica* are responsible for the majority of human disease and are strongly associated with invasive virulence [29,31,34,36,215]. Thus, it is clear that O-antigen contributes to adherence and invasion of *Yersinia* in the intestinal tract.

The canonical lipidation structure of gram-negative LPS consists of four to seven fatty acid chains, with the hexa-acylated form more commonly found in enteric bacterial pathogens. All three pathogenic *Yersiniae* synthesize hypoacylated lipid A at 37℃ compared to the hexa-acylated form at lower temperatures, as do other gram-negative bacteria [216,217]. For *Y. enterocolitica* and *Y. pestis*, hypoacylation of lipid A due to the thermal down-regulation of acylation plays an important role in mammalian virulence, likely due to the neutralization of host TLR4-recognition of LPS [218–220]. Whereas *Y. enterocolitica* expresses all lipid acylation enzymes found in *E. coli*, it also expresses LpxR, which deacylates lipid A and contributes to virulence in a mouse model of yersiniosis [221]. For *Y. pestis*, mutational inactivation of *pagP* further alters the biochemistry of lipid A [222]. PagP is an acyltransferase responsible for transferring palmitate to lipid A. Loss of PagP results in hypoacylated lipid A and consequently allows for evasion of TLR4-dependent immunity. Expression of functional PagP occurs in all known strains of *Y. enterocolitica* and *Y. pseudotuberculosis* suggesting evolutionary pressure for its retention in the gastrointestinal tract [223]. Loss of PagP appears to be an important aspect of the hyper-virulence evolution of *Y. pestis* and PagP is found in the ancient lineage strains but not those that caused the pandemics [224]. Each of these mechanisms for the production of hypoacylated lipid A allows for evasion of detection by host TLR4, thereby inherently reducing the inflammatory potential of *Yersinia* endotoxin at least in the early stage of infection.

Bioinformatics and extensive genetic data indicate that *Y. pestis* does not express flagellin due to loss of expression caused by stop codon insertion mutation into the master regulator of flagella assembly, FlhD [225]. In the other pathogenic *Yersinia spp*., flagellin expression is strongly regulated by environmental cues that result in little to no expression of flagella in the mammalian host [226]. These features indicate that mammalian TLR5, which is activated by flagella, plays no significant role in the host response to infection by pathogenic *Yersiniae*.

### Role for host inflammatory pathology during Yersinia infection

The T3SS-dependent early suppression of inflammatory cytokine production by *Y. pestis* in the lungs generates an environment that supports bacterial growth, even for avirulent species [227]. Yet the disease phase is characterized by a biphasic host inflammatory response and a profound switch to a hyper-inflammatory state [228]. A single host factor, myeloid differentiation protein 88 (MyD88), may be the key to understanding this apparent paradox. Early pulmonary stimulation of MyD88 by wild-type *Y. pestis* results in minor changes to the production of cytokines in this stage of infection, yet the environment was less permissive for colonization of the lungs [229]. Despite reduced lung colonization, however, the disease phase in *Myd88*^*-/-*^ mice was characterized by a large reduction in pulmonary inflammatory cytokines. Further evidence that MyD88 controls the hyper-inflammatory response was found in assessing pulmonary infection by non-pigmented *Y. pestis* which is unable to grow in lungs but can disseminate and cause lethal septicaemic plague [230]. In this model, loss of MyD88 resulted in increased bacterial growth in the lungs but no hyper-inflammatory response, indicating that MyD88-dependent inflammation helps control the growth of this strain and is necessary for the disease-phase hyper-inflammatory response [229]. In contrast, MyD88 May not be important during yersiniosis. In mammalian cells, two TLR adapters, MyD88 and toll/interleukin 1 receptor domain-containing adapter inducing IFNβ (TRIF), may be activated downstream of TLR4 [231]. During *Y. enterocolitica* infection of macrophages and in the mouse model, TRIF, rather than MyD88, played a role in cytokine production and host defence [232,233]. In contrast, the role of TRIF in host defence against *Y. pseudotuberculosis* may be neutralized by YopJ [234]. Although the sensitivity of mice lacking TRIF to *Y. pestis* infection has not yet been published, it appears that LPS modification and YopJ activity can be predicted to neutralize TRIF in the development of plague.

In addition, intracellular *Y. pestis* is detected by the intracellular PRR, TLR7, which is stimulated by small RNAs typically released following bacterial killing in the phagolysosome [235]. Genetic data suggest that TLR7 contributes to inflammatory pathology during plague, as mice lacking *Tlr7* were more resistant to *Y. pestis* infection with enhanced inflammatory pathology evident in the lungs and liver compared to infected wild-type mice [236]. In vitro, TLR7 contributed significantly to IFNβ production. A similar phenotype involving inflammatory pathology and resistance to infection was observed in mice lacking IFNβ signalling suggesting that TLR7-dependent type I IFN may facilitate the progression of plague [237]. Overall, it is clear that *Y. pestis* has fine-tuned its virulence to exploit the host innate immune response.

### Role of intracellular Yersinia in pathogenesis and evasion of immunity

#### Survival in activated macrophages

Pathogenic *Yersiniae* can survive inside activated macrophages where they undergo low-level replication and eventually escape through a mechanism that has not yet been clearly defined [238]. Early work established that *Yersinia* occupies an intracellular niche that is similar to an early endosome with neutral pH, the so-called *Yersinia*-containing vacuole (YCV) [239,240]. The maturation of the vacuole is halted by a PhoPQ system, with PhoQ acting as a sensor of the endosomal environment and subsequently phosphorylating PhoP, which then goes on to activate the expression of a variety of genes that contribute to bacterial survival [241,242]. Typically, only one or two bacteria occupy the spacious vacuole, with limited intracellular growth [243]. While the T3SS inhibits uptake, bacteria lacking the T3SS have no defect in intracellular survival. Instead, *Yersinia* use other mechanisms to facilitate intracellular remodelling of the phagosome in order to generate the YCV [244]. Survival in IFNγ-activated macrophages also requires the expression of metal transporters RipA, RipB, and RipC, expressed together on an operon located in the pigmentation locus [245]. The Rip (required for intracellular proliferation) proteins have been demonstrated to be required for reducing levels of nitric oxide in macrophages, assisting *Yersinia* in survival when internalized, but they likely do not play a role in remodelling the YCV [246].

The role of intracellular *Yersinia* in pathogenesis is unclear. Whereas loss of *phoP* results in intracellular killing for *Y. pestis* or *Y. pseudotuberculosis*, only the *Y. pseudotuberculosis phoP* mutant was attenuated in the mouse model [247,248]. Furthermore, intracellular killing is also observed during infection of activated macrophages or neutrophils where both outcomes (killing or survival) could likely play a role in the overall host response. For example, degradation of *Y. pestis* in a phagosome would allow detection by nucleic acid-recognizing TLRs 7 and 9 and the subsequent stimulation of NFκB and IRF-3, leading to the activation of type I IFN and inflammatory cytokine production. This would be predicted to facilitate the progression of plague, but it may stimulate bacterial clearance in the yersiniosis model.

## Autophagy

Autophagy is a highly conserved intracellular response to danger, whether it is initiated by starvation or pathogen invasion in the cytoplasm [249]. Numerous lines of evidence support a role of autophagy in innate immunity that is conserved from insects to humans. Therefore, there may be shared virulence mechanisms that target autophagy in the flea and mammalian hosts of *Y. pestis*. Autophagy is essential in mammals, but conditional knockouts have been used to screen mice for susceptibility and to examine intracellular survival in macrophages defective for autophagy during *Y. pestis* infection [243,250]. These studies suggested that autophagy plays a role in host defence rather than the promotion of pathogenesis. In contrast, *Y. pseudotuberculosis* and *Y. enterocolitica* may use autophagy to generate the YCV, as it is positive for autophagic markers, and a characteristic double membrane autophagosome has been observed during *Yersinia* infection [251,252]. However, there appears to be no anti-bacterial role for autophagy, as bacteria survive and apparently replicate in these autophagic vacuoles. Based on these data, it appears that the autophagy pathway is at least partially activated during intracellular infection and may play an important role in niche-specific host-pathogen interactions involving intracellular *Yersinia*.

## Conclusions

Pathogenic *Yersiniae* share a potent mechanism for immune evasion, the T3SS. This machinery delivers a payload of effector proteins that work together to modify the host response to infection such that innate immunity is disrupted, allowing colonization of mucosal tissues. The Yop effectors share more than 99% amino acid identity between the pathogenic *Yersinia* species indicating strong selective pressure for their collective functions. The LCR is also highly conserved as a mechanism to facilitate a heterogeneous response at the population level that facilitates rapid growth while neutralizing the phagocytic and inflammatory responses. Where these pathogens diverge relates to both gene acquisition and mutational inactivation of cell surface proteins and outer membrane structures. For *Y. pestis*, loss of function mutations appear to synergize with the acquisition of genes that facilitate survival in the flea and the transmission cycle involving rodents and their fleas. Conversely, the evolution of *Y. enterocolitica* favours its transmission through the GI route, with the acquisition of enterotoxin combined with enhanced adherence and invasion of epithelial cells facilitating transmission and disease. Therapeutic targeting of the shared mechanisms important for causing inflammatory pathology may be a useful strategy to enhance the protective efficacy of antibiotic treatments against infections caused by *Yersinia* pathogens.

## References

[1] Cui Y, Schmid B, Cao H, et al. Evolutionary selection of biofilm-mediated extended phenotypes in *Yersinia pestis* in response to a fluctuating environment. Nat Commun. 2020;11(1):281. doi: 10.1038/s41467-019-14099-w 31941912 PMC6962365

[2] Chain P, Carniel E, Larimer F, et al. Insights into the evolution of *Yersinia pestis* through whole-genome comparison with *Yersinia pseudotuberculosis*. Proc Natl Acad Sci. 2004;101(38):13826–23. doi: 10.1073/pnas.0404012101 15358858 PMC518763

[3] Rasmussen S, Allentoft ME, Nielsen K, et al. Early divergent strains of *Yersinia pestis* in Eurasia 5,000 years ago. Cell. 2015;163(3):571–82. doi: 10.1016/j.cell.2015.10.009 26496604 PMC4644222

[4] Rascovan N, Sjogren KG, Kristiansen K, et al. Emergence and spread of basal lineages of *Yersinia pestis* during the neolithic decline. Cell. 2019;176(1–2):295–305 e10. doi: 10.1016/j.cell.2018.11.005 30528431

[5] Wagner DM, Klunk J, Harbeck M, et al. *Yersinia pestis* and the plague of Justinian 541–543 AD: a genomic analysis. The Lancet Infectious Diseases. 2014;14(4):319–326. doi: 10.1016/S1473-3099(13)70323-2 24480148

[6] Tan S, Dutta A, Jakubovics N, et al. YersiniaBase: a genomic resource and analysis platform for comparative analysis of *Yersinia*. BMC Bioinformatics. 2014;16(1):9. doi: 10.1186/s12859-014-0422-y PMC4384384 25591325

[7] Duan R, Liang J, Shi G, et al. Homology analysis of pathogenic *Yersinia* species *Yersinia enterocolitica*, *Yersinia pseudotuberculosis*, and *Yersinia pestis* based on multilocus sequence typing. J Clin Microbiol. 2014;52(1):20–9. doi: 10.1128/JCM.02185-13 24131695 PMC3911470

[8] Butler T. Plague gives surprises in the first decade of the 21st century in the United States and worldwide. Am J Trop Med Hyg. 2013;89(4):788–93. doi: 10.4269/ajtmh.13-0191 24043686 PMC3795114

[9] Nelson C, Meaney-Delman D, Fleck-Derderian S, et al. Antimicrobial treatment and prophylaxis of plague: recommendations for naturally acquired infections and bioterrorism response. MMWR Recomm Rep. 2021;70(3):1–27. doi: 10.15585/mmwr.rr7003a1 PMC8312557 34264565

[10] Hinnebusch J, Cherepanov P, Du Y, et al. Murine toxin of *Yersinia pestis* shows phospholipase D activity but is not required for virulence in mice. International Journal Of Medical Microbiology. 2000;290(4–5):483–487. doi: 10.1016/S1438-4221(00)80070-3 11111930

[11] Hinnebusch B, Rudolph A, Cherepanov P, et al. Role of *Yersinia* murine toxin in survival of *Yersinia pestis* in the midgut of the flea vector. Science. 2002;296(5568):733–735. doi: 10.1126/science.1069972 11976454

[12] Hinnebusch B, Perry R, Schwan T. Role of the *Yersinia pestis* hemin storage (hms) locus in the transmission of plague by fleas. Science. 1996;273(5273):367–70. doi: 10.1126/science.273.5273.367 8662526

[13] Lindler L, Klempner M, Straley S. *Yersinia pestis* pH 6 antigen: genetic, biochemical, and virulence characterization of a protein involved in the pathogenesis of bubonic plague. Infect Immun. 1990;58(8):2569–2577. doi: 10.1128/iai.58.8.2569-2577.1990 2164509 PMC258857

[14] Dewitte A, Bouvenot T, Pierre F, et al. A refined model of how *Yersinia pestis* produces a transmissible infection in its flea vector. PLOS Pathog. 2020;16(4):e1008440. doi: 10.1371/journal.ppat.1008440 32294143 PMC7185726

[15] Bland D, Miarinjara A, Bosio C, et al. Acquisition of Yersinia murine toxin enabled *Yersinia pestis* to expand the range of mammalian hosts that sustain flea-borne plague. PloS Path. 2021;17(10):e1009995. doi: 10.1371/journal.ppat.1009995 PMC8547695 34648607

[16] Jarrett C, Deak E, Isherwood K, et al. Transmission of *Yersinia pestis* from an infectious biofilm in the flea vector. J Infect Dis. 2004;190(4):783–92. doi: 10.1086/422695 15272407

[17] Bobrov A, Kirillina O, Forman S, et al. Insights into *Yersinia pestis* biofilm development: topology and co-interaction of Hms inner membrane proteins involved in exopolysaccharide production. Env Microbiol. 2008;10(6):1419–32. doi: 10.1111/j.1462-2920.2007.01554.x 18279344

[18] Lillard J, Fetherston J, Pedersen L, et al. Sequence and genetic analysis of the hemin storage (hms) system of *Yersinia pestis*. Gene. 1997;193(1):13–21. doi: 10.1016/S0378-1119(97)00071-1 9249062

[19] Lillard J, Bearden S, Fetherston J, et al. The haemin storage (Hms+) phenotype of *Yersinia pestis* is not essential for the pathogenesis of bubonic plague in mammals. Microbiology. 1999;145(Pt 1):197–209. doi: 10.1099/13500872-145-1-197 10206699

[20] Schubert S, Rakin A, Karch H, et al. Prevalence of the “high-pathogenicity island” of *Yersinia* species among *Escherichia coli* strains that are pathogenic to humans. Infect Immun. 1998;66(2):480–5. doi: 10.1128/IAI.66.2.480-485.1998 9453599 PMC107931

[21] Hinnebusch B, Jarrett C, Bland D. Molecular and genetic mechanisms that mediate transmission of *Yersinia pestis* by fleas. Biomolecules. 2021;11(2):1–13. doi: 10.3390/biom11020210 PMC7913351 33546271

[22] Chouikha I, Hinnebusch B. Silencing urease: a key evolutionary step that facilitated the adaptation of *Yersinia pestis* to the flea-borne transmission route. Proc Natl Acad Sci. 2014;111(52):18709–14. doi: 10.1073/pnas.1413209111 25453069 PMC4284590

[23] Sun Y, Jarrett C, Bosio C, et al. Retracing the evolutionary path that led to flea-borne transmission of *Yersinia pestis*. Cell Host Microbe. 2014;15(5):578–86. doi: 10.1016/j.chom.2014.04.003 24832452 PMC4084870

[24] Fukushima H, Shimizu S, Inatsu Y. *Yersinia enterocolitica* and *Yersinia pseudotuberculosis* detection in foods. J Pathog. 2011;2011:1–9. doi: 10.4061/2011/735308 PMC3335482 22567341

[25] Grahek-Ogden D, Schimmer B, Cudjoe K, et al. Outbreak of *Yersinia enterocolitica* serogroup O: 9 infection and processed pork, Norway. Emerg Infect Dis. 2007;13(5):754–6. doi: 10.3201/eid1305.061062 17553258 PMC2738467

[26] Fukushima H, Matsuda Y, Seki R, et al. Geographical heterogeneity between far Eastern and Western countries in prevalence of the virulence plasmid, the superantigen *Yersinia pseudotuberculosis*-derived mitogen, and the high-pathogenicity island among *Yersinia pseudotuberculosis* strains. J Clin Microbiol. 2001;39(10):3541–7. doi: 10.1128/JCM.39.10.3541-3547.2001 11574570 PMC88386

[27] Saraka D, Savin C, Kouassi S, et al. *Yersinia enterocolitica*, a neglected cause of human enteric infections in Côte d’Ivoire. PloS Negl Trop Dis. 2017;11(1):e0005216. doi: 10.1371/journal.pntd.0005216 28081123 PMC5230755

[28] Scallan E, Hoekstra RM, Angulo FJ, et al. Foodborne illness acquired in the United States—Major pathogens. Emerg Infect Dis. 2011;17(1):7–15. doi: 10.3201/eid1701.P11101 21192848 PMC3375761

[29] Lucero-Estrada C, Favier G, Escudero M. An overview of *Yersinia enterocolitica* and related species in samples of different origin from San Luis, Argentina. Food Microbiol. 2020;86:103345. doi: 10.1016/j.fm.2019.103345 31703854

[30] Joutsen S, Laukkanen-Ninios R, Henttonen H, et al. *Yersinia spp*. In wild rodents and shrews in Finland. Vector Borne Zoonotic Dis. 2017;17(5):303–11. doi: 10.1089/vbz.2016.2025 28332937

[31] Duan R, Liang J, Zhang J, et al. Prevalence of *Yersinia enterocolitica* bioserotype 3/O: 3 among children with diarrhea, China, 2010–2015. Emerg Infect Dis. 2017;23(9):1502–1509. doi: 10.3201/eid2309.160827 28820132 PMC5572862

[32] Singh I, Virdi J. Production of Yersinia stable toxin (YST) and distribution of yst genes in biotype 1A strains of *Yersinia enterocolitica*. J Med Microbiol. 2004;53(11):1065–8. doi: 10.1099/jmm.0.45527-0 15496381

[33] Delor I, Kaeckenbeeck A, Wauters G, et al. Nucleotide sequence of yst, the *Yersinia enterocolitica* gene encoding the heat-stable enterotoxin, and prevalence of the gene among pathogenic and nonpathogenic *Yersiniae*. Infect Immun. 1990;58:2983–2988. doi: 10.1128/iai.58.9.2983-2988.1990 2201642 PMC313599

[34] Bengoechea J, Najdenski H, Skurnik M. Lipopolysaccharide O antigen status of *Yersinia enterocolitica* O: 8 is essential for virulence and absence of O antigen affects the expression of other *Yersinia* virulence factors. Mol Microbiol. 2004;52(2):451–69. doi: 10.1111/j.1365-2958.2004.03987.x 15066033

[35] Skurnik M, Venho R, Bengoechea J, et al. The lipopolysaccharide outer core of *Yersinia enterocolitica* serotype O: 3 is required for virulence and plays a role in outer membrane integrity. Mol Microbiol. 1999;31(5):1443–62. doi: 10.1046/j.1365-2958.1999.01285.x 10200964

[36] Al-Hendy A, Toivanen P, Skurnik M. Lipopolysaccharide O side chain of *Yersinia enterocolitica* O: 3 is an essential virulence factor in an orally infected murine model. Infect Immun. 1992;60(3):870–5. doi: 10.1128/iai.60.3.870-875.1992 1311707 PMC257567

[37] European Food Safety A, European Centre for Disease P, Control. The European Union summary report on trends and sources of zoonoses, zoonotic agents and food-borne outbreaks in 2017. EFSA J. 2018;16(12):e05500. doi: 10.2903/j.efsa.2018.5500 32625785 PMC7009540

[38] Liang J, Wang X, Xiao Y, et al. Prevalence of *Yersinia enterocolitica* in pigs slaughtered in Chinese abattoirs. Appl Environ Microbiol. 2012;78(8):2949–56. doi: 10.1128/AEM.07893-11 22327599 PMC3318836

[39] Riahi SM, Ahmadi E, Zeinali T. Global prevalence of *Yersinia enterocolitica* in cases of gastroenteritis: a systematic review and meta-analysis. Int J Microbiol. 2021;2021:1499869. doi: 10.1155/2021/1499869 34512763 PMC8433020

[40] Durand E, Maldonado-Arocho F, Castillo C, et al. The presence of professional phagocytes dictates the number of host cells targeted for Yop translocation during infection. Cell Microbiol. 2010;12(8):1064–82. doi: 10.1111/j.1462-5822.2010.01451.x 20148898 PMC2906667

[41] Sebbane F, Uversky V, Anisimov A. *Yersinia pestis* plasminogen activator. Biomolecules. 2020;10(11):1554. doi: 10.3390/biom10111554 33202679 PMC7696990

[42] Sodeinde O, Subrahmanyam Y, Stark K, et al. A surface protease and the invasive character of plague. Science. 1992;258(5084):1004–7. doi: 10.1126/science.1439793 1439793

[43] Satala D, Bednarek A, Kozik A, et al. The recruitment and activation of plasminogen by bacteria—the involvement in chronic infection development. IJMS. 2023;24(13):24. doi: 10.3390/ijms241310436 PMC10341603 37445613

[44] Heissig B, Salama Y, Takahashi S, et al. The multifaceted role of plasminogen in inflammation. Cell Signal. 2020;75:109761. doi: 10.1016/j.cellsig.2020.109761 32861744 PMC7452830

[45] Galvan E, Lasaro M, Schifferli D. Capsular antigen fraction 1 and Pla modulate the susceptibility of *Yersinia pestis* to pulmonary antimicrobial peptides such as cathelicidin. Infect Immun. 2008;76(4):1456–1464. doi: 10.1128/IAI.01197-07 18227173 PMC2292867

[46] Liu F, Chen H, Galvan E, et al. Effects of psa and F1 on the adhesive and invasive interactions of *Yersinia pestis* with human respiratory tract epithelial cells. Infect Immun. 2006;74(10):5636–5644. doi: 10.1128/IAI.00612-06 16988239 PMC1594889

[47] Cowan C, Jones H, Kaya Y, et al. Invasion of epithelial cells by *Yersinia pestis*: evidence for a *Y.pestis* -specific invasin. Infect Immun. 2000;68(8):4523–4530. doi: 10.1128/IAI.68.8.4523-4530.2000 10899851 PMC98364

[48] Banerjee S, Crane S, Pechous R. A dual role for the plasminogen activator protease during the preinflammatory phase of primary pneumonic plague. J Infect Dis. 2020;222(3):407–16. doi: 10.1093/infdis/jiaa094 32128567 PMC7336565

[49] Lahteenmaki K, Kukkonen M, Korhonen T. The Pla surface protease/adhesin of *Yersinia pestis* mediates bacterial invasion into human endothelial cells. FEBS Lett. 2001;504(1–2):69–72. doi: 10.1016/S0014-5793(01)02775-2 11522299

[50] Kumar A, Harjai K, Chhibber S. Early cytokine response to lethal challenge of *Klebsiella pneumoniae* averted the prognosis of pneumonia in FyuA immunized mice. Microb Pathog. 2020;144:104161. doi: 10.1016/j.micpath.2020.104161 32194179

[51] Rakin A, Heesemann J. Virulence-associated fyuA / irp2 gene cluster of *Yersinia enterocolitica* biotype 1B carries a novel insertion sequence is 1328. FEMS Microbiology Letters. 1994;129(2–3):287–292. doi: 10.1111/j.1574-6968.1995.tb07594.x 7607411

[52] Zeth K. Structure and uptake mechanism of bacteriocins targeting peptidoglycan renewal. Biochem Soc Trans. 2012;40(6):1560–5. doi: 10.1042/BST20120194 23176517

[53] Hall P, Brubaker R. Pesticin-dependent generation of osmotically stable spheroplast-like structures. J Bacteriol. 1978;136(2):786–789. doi: 10.1128/jb.136.2.786-789.1978 361722 PMC218605

[54] Vollmer W, Pilsl H, Hantke K, et al. Pesticin displays muramidase activity. J Bacteriol. 1997;179(5):1580–3. doi: 10.1128/jb.179.5.1580-1583.1997 9045816 PMC178869

[55] Achtman M, Morelli G, Zhu P, et al. Microevolution and history of the plague bacillus, *Yersinia pestis*. Proc Natl Acad Sci. 2004;101(51):17837–42. doi: 10.1073/pnas.0408026101 15598742 PMC535704

[56] Eppinger M, Worsham P, Nikolich M, et al. Genome sequence of the deep-rooted *Yersinia pestis* strain Angola reveals new insights into the evolution and pangenome of the plague bacterium. J Bacteriol. 2010;192(6):1685–99. doi: 10.1128/JB.01518-09 20061468 PMC2832528

[57] Leal N, Sobreira M, Araujo A, et al. Viability of *Yersinia pestis* subcultures in agar stabs. Lett Appl Microbiol. 2016;62(1):91–5. doi: 10.1111/lam.12519 26524218

[58] Lindler L, Plano G, Burland V, et al. Complete DNA sequence and detailed analysis of the *Yersinia pestis* KIM5 plasmid encoding murine toxin and capsular antigen. Infect Immun. 1998;66(12):5731–42. doi: 10.1128/IAI.66.12.5731-5742.1998 9826348 PMC108724

[59] Zavialov A, Berglund J, Pudney A, et al. Structure and biogenesis of the capsular F1 antigen from *Yersinia pestis*: preserved folding energy drives fiber formation. Cell. 2003;113(5):587–96. doi: 10.1016/S0092-8674(03)00351-9 12787500

[60] Peters D, Reifs A, Alonso-Caballero A, et al. Unraveling the molecular determinants of the anti-phagocytic protein cloak of plague bacteria. PLOS Pathog. 2022;18(3):e1010447. doi: 10.1371/journal.ppat.1010447 35358289 PMC9004762

[61] Friedlander A, Welkos S, Worsham P, et al. Relationship between virulence and immunity as revealed in recent studies of the Fl capsule of *Yersinia pestis*. Clinical Infectious Diseases. 1995;21(Supplement_2):S178–81. doi: 10.1093/clinids/21.Supplement_2.S178 8845449

[62] Anderson G, Worsham P, Bolt C, et al. Protection of mice from fatal bubonic and pneumonic plague by passive immunization with monoclonal antibodies against the F1 protein of *Yersinia pestis*. The American Journal Of Tropical Medicine And Hygiene. 1997;56(4):471–473. doi: 10.4269/ajtmh.1997.56.471 9158060

[63] Kolodziejek A, Hovde C, Minnich S. Contributions of *Yersinia pestis* outer membrane protein ail to plague pathogenesis. Curr Opin Infect Dis. 2022;35(3):188–95. doi: 10.1097/QCO.0000000000000830 35665712 PMC9186061

[64] Bartra S, Styer K, O’Bryant D, et al. Resistance of *Yersinia pestis* to complement-dependent killing is mediated by the Ail outer membrane protein. Infect Immun. 2008;76(2):612–22. doi: 10.1128/IAI.01125-07 18025094 PMC2223467

[65] Muhlenkamp M, Oberhettinger P, Leo J, et al. *Yersinia* adhesin a (YadA) – beauty & beast. International Journal Of Medical Microbiology. 2015;305(2):252–258. doi: 10.1016/j.ijmm.2014.12.008 25604505

[66] Chauhan N, Wrobel A, Skurnik M, et al. *Yersinia* adhesins: an arsenal for infection. Proteomics Clin Appl. 2016;10(9–10):949–63. doi: 10.1002/prca.201600012 27068449

[67] Thomson J, Plecha S, Krukonis E. Ail provides multiple mechanisms of serum resistance to *Yersinia pestis*. Mol Microbiol. 2019;111(1):82–95. doi: 10.1111/mmi.14140 30260060 PMC6351204

[68] Tsang T, Felek S, Krukonis E. Ail binding to fibronectin facilitates *Yersinia pestis* binding to host cells and Yop delivery. Infect Immun. 2010;78(8):3358–68. doi: 10.1128/IAI.00238-10 20498264 PMC2916272

[69] Tsang T, Wiese J, Felek S, et al. Ail proteins of *Yersinia pestis* and *Y. pseudotuberculosis* have different cell binding and invasion activities. PloS One. 2013;8(12):e83621. doi: 10.1371/journal.pone.0083621 24386237 PMC3873954

[70] Skurnik M, Wolf-Watz H. Analysis of the yopA gene encoding the Yop1 virulence determinants of *Yersinia* spp. Mol Microbiol. 1989;3(4):517–29. doi: 10.1111/j.1365-2958.1989.tb00198.x 2761389

[71] Paczosa M, Fisher M, Maldonado-Arocho F, et al. *Yersinia pseudotuberculosis* uses ail and YadA to circumvent neutrophils by directing Yop translocation during lung infection. Cell Microbiol. 2014;16(2):247–68. doi: 10.1111/cmi.12219 24119087 PMC3981955

[72] China B, N’Guyen B, de Bruyere M, et al. Role of YadA in resistance of *Yersinia enterocolitica* to phagocytosis by human polymorphonuclear leukocytes. Infect Immun. 1994;62(4):1275–81. doi: 10.1128/iai.62.4.1275-1281.1994 8132334 PMC186269

[73] Kirjavainen V, Jarva H, Biedzka-Sarek M, et al. *Yersinia enterocolitica* serum resistance proteins YadA and ail bind the complement regulator C4b-binding protein. PLOS Pathog. 2008;4(8):e1000140. doi: 10.1371/journal.ppat.1000140 18769718 PMC2516929

[74] Casutt-Meyer S, Renzi F, Schmaler M, et al. Oligomeric coiled-coil adhesin YadA is a double-edged sword. PloS One. 2010;5(12):e15159. doi: 10.1371/journal.pone.0015159 21170337 PMC2999546

[75] Gillenius E, Urban C. The adhesive protein invasin of *Yersinia pseudotuberculosis* induces neutrophil extracellular traps via β1 integrins. Microbes And Infection. 2015;17(5):327–336. doi: 10.1016/j.micinf.2014.12.014 25576025

[76] Pepe J, Badger J, Miller V. Growth phase and low pH affect the thermal regulation of the *Yersinia enterocolitica* inv gene. Mol Microbiol. 1994;11(1):123–35. doi: 10.1111/j.1365-2958.1994.tb00295.x 7511772

[77] Uliczka F, Pisano F, Schaake J, et al. Unique cell adhesion and invasion properties of *Yersinia enterocolitica* O: 3, the most frequent cause of human yersiniosis. PLOS Pathog. 2011;7(7):e1002117. doi: 10.1371/journal.ppat.1002117 21750675 PMC3131269

[78] Grassl G, Bohn E, Muller Y, et al. Interaction of *Yersinia enterocolitica* with epithelial cells: invasin beyond invasion. Int J Med Microbiol. 2003;293(1):41–54. doi: 10.1078/1438-4221-00243 12755365

[79] Yang Y, Isberg R. Cellular internalization in the absence of invasin expression is promoted by the *Yersinia pseudotuberculosis* yadA product. Infect Immun. 1993;61(9):3907–13. doi: 10.1128/iai.61.9.3907-3913.1993 8359913 PMC281093

[80] Pepe J, Miller V. *Yersinia enterocolitica* invasin: a primary role in the initiation of infection. Proc Natl Acad Sci U S A. 1993;90(14):6473–7. doi: 10.1073/pnas.90.14.6473 8341658 PMC46954

[81] Fasciano A, Dasanayake G, Estes M, et al. *Yersinia pseudotuberculosis* YopE prevents uptake by M cells and instigates M cell extrusion in human ileal enteroid-derived monolayers. Gut Microbes. 2021;13(1):1988390. doi: 10.1080/19490976.2021.1988390 34793276 PMC8604394

[82] Rosqvist R, Forsberg A, Rimpilainen M, et al. The cytotoxic protein YopE of *Yersinia* obstructs the primary host defence. Molecular Microbiology. 1990;4(4):657–667. doi: 10.1111/j.1365-2958.1990.tb00635.x 2191183

[83] Cornelis G. *Yersinia* type III secretion: send in the effectors. J Cell Bio. 2002;158(3):401–8. doi: 10.1083/jcb.200205077 12163464 PMC2173816

[84] Mehigh R, Sample A, Brubaker R. Expression of the low calcium response in *Yersinia pestis*. Microb Pathog. 1989;6(3):203–17. doi: 10.1016/0882-4010(89)90070-3 2739560

[85] Yother J, Chamness T, Goguen J. Temperature-controlled plasmid regulon associated with low calcium response in *Yersinia pestis*. J Bacteriol. 1986;165(2):443–7. doi: 10.1128/jb.165.2.443-447.1986 3944056 PMC214438

[86] Goguen J, Yother J, Straley S. Genetic analysis of the low calcium response in *Yersinia pestis* mu d1 (ap *lac)* insertion mutants. J Bacteriol. 1984;160(3):842–8. doi: 10.1128/jb.160.3.842-848.1984 6094509 PMC215785

[87] Plano G, Straley S. Mutations in *yscC*, *yscD*, and *yscG* prevent high-level expression and secretion of V antigen and yops in *Yersinia pestis*. J Bacteriol. 1995;177(13):3843–54. doi: 10.1128/jb.177.13.3843-3854.1995 7601852 PMC177105

[88] Fowler J, Wulff C, Straley S, et al. Growth of calcium-blind mutants of *Yersinia pestis* at 37 °C in permissive Ca2+-deficient environments. Microbiology (Reading). 2009;155(8):2509–2521. doi: 10.1099/mic.0.028852-0 19443541 PMC2888125

[89] Yother J, Goguen J. Isolation and characterization of Ca2^+^blind mutants of *Yersinia pestis*. J Bacteriol. 1985;164(2):704–711. doi: 10.1128/jb.164.2.704-711.1985 2997127 PMC214309

[90] Perry R, Brubaker R. Vwa+ phenotype of *Yersinia enterocolitica*. Infect Immun. 1983;40(1):166–71. doi: 10.1128/iai.40.1.166-171.1983 6832830 PMC264831

[91] Marketon M, DePaolo R, DeBord K, et al. Plague bacteria target immune cells during infection. Science. 2005;309(5741):1739–41. doi: 10.1126/science.1114580 16051750 PMC3210820

[92] Bohme K, Steinmann R, Kortmann J, et al. Concerted actions of a thermo-labile regulator and a unique intergenic RNA thermosensor control *Yersinia* virulence. PLOS Pathog. 2012;8(2):e1002518. doi: 10.1371/journal.ppat.1002518 22359501 PMC3280987

[93] Hoe N, Goguen J. Temperature sensing in *Yersinia pestis*: translation of the LcrF activator protein is thermally regulated. J Bacteriol. 1993;174(24):7901–7909. doi: 10.1128/jb.175.24.7901-7909.1993 PMC206968 7504666

[94] Schwiesow L, Lam H, Dersch P, et al. *Yersinia* type III secretion system master regulator lcrF. J Bacteriol. 2015;198(4):604–614. doi: 10.1128/JB.00686-15 26644429 PMC4751813

[95] Garrity-Ryan L, Kim O, Balada-Llasat J, et al. Small molecule inhibitors of LcrF, a *Yersinia pseudotuberculosis* transcription factor, attenuate virulence and limit infection in a murine pneumonia model. Infect Immun. 2010;78(11):4683–90. doi: 10.1128/IAI.01305-09 20823209 PMC2976336

[96] King J, Schesser Bartra S, Plano G, et al. ExsA and LcrF recognize similar consensus binding sites, but differences in their oligomeric state influence interactions with promoter DNA. J Bacteriol. 2013;195(24):5639–50. doi: 10.1128/JB.00990-13 24142246 PMC3889609

[97] Jackson M, Silva-Herzog E, Plano G. The ATP-dependent ClpXP and lon proteases regulate expression of the *Yersinia pestis* type III secretion system via regulated proteolysis of YmoA, a small histone-like protein. Mol Microbiol. 2004;54(5):1364–78. doi: 10.1111/j.1365-2958.2004.04353.x 15554975

[98] Rohde J, Fox J, Minnich S. Thermoregulation in *Yersinia enterocolitica* is coincident with changes in DNA supercoiling. Mol Microbiol. 1994;12(2):187–99. doi: 10.1111/j.1365-2958.1994.tb01008.x 8057844

[99] Worrall L, Majewski D, Strynadka N. Type III secretion systems of the bacterial flagellum and injectisome. Ann Rev Microbiol. 2023;77(1):669–698. doi: 10.1146/annurev-micro-032521-025503 37713458

[100] Mueller C, Broz P, Muller S, et al. The V-Antigen of *Yersinia* forms a distinct structure at the tip of injectisome needles. Science. 2005;310(5748):674–676. doi: 10.1126/science.1118476 16254184

[101] Hayes C, Aoki S, Low D. Bacterial contact-dependent delivery systems. Ann Rev Genet. 2010;44(1):71–90. doi: 10.1146/annurev.genet.42.110807.091449 21047256

[102] Journet L, Agrain C, Broz P, et al. The needle length of bacterial injectisomes is determined by a molecular ruler. Science. 2003;302(5651):1757–60. doi: 10.1126/science.1091422 14657497

[103] Payne P, Straley S. YscP of *Yersinia pestis* is a secreted component of the yop secretion system. J Bacteriol. 1999;181(9):2852–62. doi: 10.1128/JB.181.9.2852-2862.1999 10217778 PMC93729

[104] Agrain C, Sorg I, Paroz C, et al. Secretion of YscP from *Yersinia enterocolitica* is essential to control the length of the injectisome needle but not to change the type III secretion substrate specificity. Mol Microbiol. 2005;57(5):1415–27. doi: 10.1111/j.1365-2958.2005.04758.x 16102009

[105] Bjornfot A, Lavander M, Forsberg A, et al. Autoproteolysis of YscU of *Yersinia pseudotuberculosis* is important for regulation of expression and secretion of Yop proteins. J Bacteriol. 2009;191(13):4259–4267. doi: 10.1128/JB.01730-08 19395493 PMC2698497

[106] Williams A, Straley S. YopD of *Yersinia pestis* plays a role in negative regulation of the low-calcium response in addition to its role in translocation of yops. J Bacteriol. 1998;180(2):350–358. doi: 10.1128/JB.180.2.350-358.1998 9440524 PMC106890

[107] Anderson D, Ramamurthi K, Tam C, et al. YopD and LcrH regulate expression of *Yersinia enterocolitica* YopQ by a posttranscriptional mechanism and bind to *yopQ* RNA. J Bacteriol. 2002;184(5):1287–95. doi: 10.1128/JB.184.5.1287-1295.2002 11844757 PMC134855

[108] Chen Y, Anderson D. Expression hierarchy in the *Yersinia* type III secretion system established through YopD recognition of RNA. Mol Microbiol. 2011;80(4):966–80. doi: 10.1111/j.1365-2958.2011.07623.x 21481017 PMC4128491

[109] Anderson D, Scheewind O. A mRNA signal for the type III secretion of Yop proteins by *Yersinia enterocolitica*. Science. 1997;278(5340):1140–1143. doi: 10.1126/science.278.5340.1140 9353199

[110] Anderson D, Schneewind O. *Yersinia enterocolitica* type III secretion: an mRNA signal that couples translation and secretion of YopQ. Mol Microbiol. 1999;31(4):1139–48. doi: 10.1046/j.1365-2958.1999.01254.x 10096081

[111] Rimpilainen M, Forsberg A, Wolf-Watz H. A novel protein, LcrQ, involved in the low-calcium response of *Yersinia pseudotuberculosis* shows extensive homology to YopH. J Bacteriol. 1992;174(10):3355–63. doi: 10.1128/jb.174.10.3355-3363.1992 1577700 PMC206005

[112] Cambronne E, Cheng L, Schneewind O. LcrQ/YscM1, regulators of the *Yersinia yop* virulon, are injected into host cells by a chaperone-dependent mechanism. Mol Microbiol. 2000;37(2):263–73. doi: 10.1046/j.1365-2958.2000.01974.x 10931323

[113] Wulff-Strobel C, Williams A, Straley S. LcrQ and SycH function together at the ysc type III secretion system in *Yersinia pestis* to impose a hierarchy of secretion. Mol Microbiol. 2002;43(2):411–23. doi: 10.1046/j.1365-2958.2002.02752.x 11985718

[114] Petersson J, Nordfelth R, Dubinina E, et al. Modulation of virulence factor expression by pathogen target cell contact. Science. 1996;273(5279):1183–1184. doi: 10.1126/science.273.5279.1231 8703058

[115] Li Y, Li D, Shao H, et al. Plague in China 2014—all sporadic case report of pneumonic plague. BMC Infect Dis. 2016;16(1):1–8. doi: 10.1186/s12879-016-1403-8 26895880 PMC4759734

[116] Hoiczyk E, Blobel G. Polymerization of a single protein of the pathogen *Yersinia enterocolitica* into needles punctures eukaryotic cells. Proc Natl Acad Sci U S A. 2001;98(8):4669–74. doi: 10.1073/pnas.071065798 11287645 PMC31892

[117] Rudolph M, Carsten A, Kulnik S, et al. Live imaging of *Yersinia* translocon formation and immune recognition in host cells. PloS Path. 2022;18(5):e1010251. doi: 10.1371/journal.ppat.1010251 PMC9173619 35604950

[118] Weeks S, Hill J, Friedlander A, et al. Anti-V antigen antibody protects macrophages from *Yersinia pestis*-induced cell death and promotes phagocytosis. Microb Path. 2002;32(5):227–37. doi: 10.1006/mpat.2002.0498 12071679

[119] Sarker M, Neyt C, Stainier I, et al. The *Yersinia* Yop virulon: LcrV is required for extrusion of the translocators YopB and YopD. J Bacteriol. 1998;180(5):1207–14. doi: 10.1128/JB.180.5.1207-1214.1998 9495760 PMC107009

[120] Hakansson S, Bergman T, Vanooteghem J, et al. YopB and YopD constitute a novel class of *Yersinia* Yop proteins. Infect Immun. 1993;61(1):71–80. doi: 10.1128/iai.61.1.71-80.1993 8418066 PMC302689

[121] Hakansson S, Schesser K, Persson C, et al. The YopB protein of *Yersinia pseudotuberculosis* is essential for the translocation of Yop effector proteins across the target cell plasma membrane and displays a contact-dependent membrane disrupting activity. EMBO J. 1996;15(21):5812–23. doi: 10.1002/j.1460-2075.1996.tb00968.x 8918459 PMC452329

[122] Nordfelth R, Wolf-Watz H, Finlay BB. YopB of *Yersinia enterocolitica* is essential for YopE translocation. Infect Immun. 2001;69(5):3516–8. doi: 10.1128/IAI.69.5.3516-3518.2001 11292787 PMC98323

[123] Costa T, Amer A, Farag S, et al. Type III secretion translocon assemblies that attenuate *Yersinia* virulence. Cell Microbiol. 2013;15(7):1088–110. doi: 10.1111/cmi.12100 23279117

[124] Osei-Owusu P, Charlton T, Kim H, et al. FPR1 is the plague receptor on host immune cells. Nature. 2019;574(57):62. doi: 10.1038/s41586-019-1570-z PMC6776691 31534221

[125] Yang D, Chen Q, Le Y, et al. Differential regulation of formyl peptide receptor-like 1 expression during the differentiation of monocytes to dendritic cells and macrophages. J Immunol. 2001;166(6):4092–8. doi: 10.4049/jimmunol.166.6.4092 11238658

[126] Daniel C, Dewitte A, Poiret S, et al. Polymorphism in the *Yersinia* LcrV antigen enables immune escape from the protection conferred by an LcrV-secreting *Lactococcus lactis* in a pseudotuberculosis mouse model. Front Immunol. 2019;10:1830. doi: 10.3389/fimmu.2019.01830 31428104 PMC6688116

[127] DiMezzo T, Ruthel G, Brueggemann E, et al. *In vitro* intracellular trafficking of virulence antiben during infection by *Yersinia pestis*. PloS One. 2009;4(7):e6281. doi: 10.1371/journal.pone.0006281 19609450 PMC2707630

[128] Fields K, Straley S, Burns DL. LcrV of *Yersinia pestis* enters infected eukaryotic cells by a virulence plasmid-independent mechanism. Infect Immun. 1999;67(9):4801–4813. doi: 10.1128/IAI.67.9.4801-4813.1999 10456934 PMC96812

[129] Godlee C, Holden D. Transmembrane substrates of type three secretion system injectisomes. Micobiol. 2023;169(1):001292. doi: 10.1099/mic.0.001292 PMC9993115 36748571

[130] Brodsky I, Palm N, Sadanand S, et al. A */Yersinia* effector protein promotes virulence by preventing inflammasome recognition of the type III secretion system. Cell Host Microbe. 2010;7(5):376–87. doi: 10.1016/j.chom.2010.04.009 20478539 PMC2883865

[131] Trosky J, Liverman A, Orth K. *Yersinia* outer proteins: Yops. Cell Microbiol. 2008;10(3):557–65. doi: 10.1111/j.1462-5822.2007.01109.x 18081726

[132] Pha K, Navarro L. *Yersinia* type III effectors perturb host innate immune responses. World J Biol Chem. 2016;7(1):1–13. doi: 10.4331/wjbc.v7.i1.1 26981193 PMC4768113

[133] Ryndak M, Chung H, London E, et al. Role of predicted transmembrane domains for type III translocation, pore formation, and signaling by the *Yersinia pseudotuberculosis* YopB protein. Infect Immun. 2005;73(4):2433–43. doi: 10.1128/IAI.73.4.2433-2443.2005 15784589 PMC1087397

[134] Mattei P, Faudry E, Job V, et al. Membrane targeting and pore formation by the type III secretion system translocon. FEBS J. 2011;278(3):414–26. doi: 10.1111/j.1742-4658.2010.07974.x 21182592

[135] Guichon A, Hersh D, Smith M, et al. Structure-function analysis of the *Shigella* virulence factor IpaB. J Bacteriol. 2001;183(4):1269–76. doi: 10.1128/JB.183.4.1269-1276.2001 11157939 PMC95000

[136] Sarhan J, Liu B, Muendlein H, et al. Caspase-8 induces cleavage of gasdermin D to elicit pyroptosis during *Yersinia* infection. Proc Natl Acad Sci. 2018;115(46):e10888–e97. doi: 10.1073/pnas.1809548115 30381458 PMC6243247

[137] DeBord K, Lee V, Schneewind O. Roles of LcrG and LcrV during type III targeting of effector yops by *Yersinia enterocolitica*. J Bacteriol. 2001;183(15):4588–98. doi: 10.1128/JB.183.15.4588-4598.2001 11443094 PMC95354

[138] Dewoody R, Merritt P, Marketon M. YopK controls both rate and fidelity of Yop translocation. Mol Microbiol. 2013;87(2):301–17. doi: 10.1111/mmi.12099 23205707 PMC3545096

[139] Veenendaal A, Hodgkinson J, Schwarzer L, et al. The type III secretion system needle tip complex mediates host cell sensing and translocon insertion. Mol Microbiol. 2007;63(6):1719–30. doi: 10.1111/j.1365-2958.2007.05620.x 17367391

[140] Straley S, Cibull M. Differential clearance and host-pathogen interactions of YopE- and YopK-YopL- *Yersinia pestis* in BALB/c mice. Infect Immun. 1989;57(4):1200–10. doi: 10.1128/iai.57.4.1200-1210.1989 2925246 PMC313251

[141] Logsdon L, Mecsas J. Requirement of the *Yersinia pseudotuberculosis* effectors YopH and YopE in colonization and persistence in intestinal and lymph tissues. Infect Immun. 2003;71(8):4595–607. doi: 10.1128/IAI.71.8.4595-4607.2003 12874339 PMC166012

[142] Hall A. Rho family GTPases. Biochem Soc Trans. 2012;40(6):1378–82. doi: 10.1042/BST20120103 23176484

[143] Rosqvist R, Forsberg A, Wolf-Watz H. Intracellular targeting of the *Yersinia* YopE cytotoxin in mammalian cells induces actin microfilament disruption. Infect Immun. 1991;59(12):4562–9. doi: 10.1128/iai.59.12.4562-4569.1991 1937815 PMC259078

[144] Black D, Bliska J. The RhoGAP activity of the *Yersinia pseudotuberculosis* cytotoxin YopE is required for antiphagocytic function and virulence. Mol Microbiol. 2000;37(3):515–27. doi: 10.1046/j.1365-2958.2000.02021.x 10931345

[145] Van Aelst L, D’Souza-Schorey C. Rho GTPases and signaling networks. Genes Dev. 1997;11(18):2295–322. doi: 10.1101/gad.11.18.2295 9308960

[146] Aepfelbacher M, Heesemann J. Modulation of rho GTPases and the actin cytoskeleton by *Yersinia* outer proteins (yops). Int J Med Microbiol. 2001;291(4):269–76. doi: 10.1078/1438-4221-00130 11680787

[147] Medici N, Rashid M, Bliska J, et al. Characterization of pyrin dephosphorylation and inflammasome activation in macrophages as triggered by the *Yersinia* effectors YopE and YopT. Infect Immun. 2019;87(3). doi: 10.1128/IAI.00822-18 PMC6386549 30602502

[148] Trulzsch K, Sporleder T, Igwe E, et al. Contribution of the major secreted yops of *Yersinia enterocolitica* O: 8 to pathogenicity in the mouse infection model. Infect Immun. 2004;72(9):5227–5234. doi: 10.1128/IAI.72.9.5227-5234.2004 15322017 PMC517446

[149] Cantwell A, Bubeck S, Dube P. YopH inhibits early pro-inflammatory cytokine responses during plague pneumonia. BMC Immunol. 2010;11(1):29–40. doi: 10.1186/1471-2172-11-29 20565713 PMC2894752

[150] Zhang Y, Bliska J. Role of toll-like receptor signaling in the apoptotic response of macrophages to *Yersinia* infection. Infect Immun. 2003;71(3):1513–9. doi: 10.1128/IAI.71.3.1513-1519.2003 12595470 PMC148878

[151] Guan K, Dixon J. Bacterial and viral protein tyrosine phosphatases. Sem Cell Biol. 1993;4(6):389–396. doi: 10.1006/scel.1993.1046 8305677

[152] Fallman M, Deleuil F, McGee K. Resistance to phagocytosis by *Yersinia*. International Journal Of Medical Microbiology. 2002;291(6–7):501–509. doi: 10.1078/1438-4221-00159 11890550

[153] Andersson K, Carballeira N, Magnussen K, et al. YopH of *Yersinia pseudotuberculosis* interrupts early phosphotyrosine signalling associated with phagocytosis. Molecular Microbiology. 1996;20(5):1057–1069. doi: 10.1111/j.1365-2958.1996.tb02546.x 8809758

[154] Hamid N, Gustavsson A, Andersson K, et al. YopH dephosphorylates cas and fyn-binding protein in macrophages. Microb Pathog. 1999;27(4):231–42. doi: 10.1006/mpat.1999.0301 10502464

[155] Persson C, Carballeira N, Wolf-Watz H, et al. The PTPase YopH inhibits uptake of *Yersinia*, tyrosine phosphorylation of p130Cas and FAK, and the associated accumulation of these proteins in peripheral focal adhesions. EMBO J. 1997;16(9):2307–18. doi: 10.1093/emboj/16.9.2307 9171345 PMC1169832

[156] Persson C, Nordfelth R, Andersson K, et al. Localization of the *Yersinia* PTPase to focal complexes is an important virulence mechanism. Molecular Microbiology. 1999;33(4):828–838. doi: 10.1046/j.1365-2958.1999.01529.x 10447891

[157] Rolan H, Durand E, Mecsas J. Identifying *Yersinia* YopH-targeted signal transduction pathways that impair neutrophil responses during in vivo murine infection. Cell Host Microbe. 2013;14(3):306–17. doi: 10.1016/j.chom.2013.08.013 24034616 PMC3789382

[158] Ruckdeschel K, Mannel O, Richter K, et al. Yersinia outer protein p of *Yersinia enterocolitica* simultaneously blocks the nuclear factor-κB pathway and exploits lipopolysaccharide signaling to trigger apoptosis in macrophages. The Journal Of Immunology. 2001;166(3):1823–1831. doi: 10.4049/jimmunol.166.3.1823 11160229

[159] Orth K. Function of the *Yersinia* effector YopJ. Curr Opin Microbiol. 2002;5(1):38–43. doi: 10.1016/S1369-5274(02)00283-7 11834367

[160] Zhou H, Monack D, Kayagaki N, et al. *Yersinia* virulence factor YopJ acts as a deubiquitinase to inhibit NF-κB activation. The Journal Of Experimental Medicine. 2005;202(10):1327–1332. doi: 10.1084/jem.20051194 16301742 PMC2212976

[161] Orth K, Xu Z, Mudgett M, et al. Disruption of signaling by *Yersinia* effector YopJ, a ubiquitin-like protein protease. Science. 2000;290(5496):1594–7. doi: 10.1126/science.290.5496.1594 11090361

[162] Ma K, Ma W. YopJ family effectors promote bacterial infection through a unique acetyltransferase activity. Microbiol Mol Biol Rev. 2016;80(4):1011–27. doi: 10.1128/MMBR.00032-16 27784797 PMC5116873

[163] Sweet C, Conlon J, Golenbock D, et al. YopJ targets TRAF proteins to inhibit TLR-mediated NF-kB, MAPK and IRF3 signal transduction. Cell Microbiol. 2007;9(11):2700–2715. doi: 10.1111/j.1462-5822.2007.00990.x 17608743

[164] Bliska J. *Yersinia* inhibits host signaling by acetylating MAPK kinases. ACS Chem Biol. 2006;1(6):349–51. doi: 10.1021/cb600261k 17163770

[165] Monack D, Mecsas J, Ghori N, et al. *Yersinia* signals macrophages to undergo apoptosis and YopJ is necessary for this cell death. Proc Natl Acad Sci. 1997;94(19):10385–90. doi: 10.1073/pnas.94.19.10385 9294220 PMC23372

[166] Orning P, Weng D, Starheim K, et al. Pathogen blockade of TAK1 triggers caspase-8–dependent cleavage of gasdermin D and cell death. Science. 2018;362(6418):1064–1069. doi: 10.1126/science.aau2818 30361383 PMC6522129

[167] Philip N, Brodsky I. Cell death programs in *Yersinia* immunity and pathogenesis. Front Cell Infect Microbiol. 2012;2:149. doi: 10.3389/fcimb.2012.00149 23226685 PMC3510641

[168] Shao F, Vacratsis P, Bao Z, et al. Biochemical characterization of the *Yersinia* YopT protease: cleavage site and recognition elements in rho GTPases. Proc Natl Acad Sci. 2003;100(3):904–9. doi: 10.1073/pnas.252770599 12538863 PMC298699

[169] Iriarte M, Cornelis G. YopT, a new *Yersinia* Yop effector protein, affects the cytoskeleton of host cells. Mol Microbiol. 1998;29(3):915–29. doi: 10.1046/j.1365-2958.1998.00992.x 9723929

[170] Wong K, Isberg R, Galan J. *Yersinia pseudotuberculosis* spatially controls activation and misregulation of host cell Rac1. PLOS Pathog. 2005;1(2):e16. doi: 10.1371/journal.ppat.0010016 16228016 PMC1253843

[171] Viboud G, Mejia E, Bliska J. Comparison of YopE and YopT activities in counteracting host signalling responses to *Yersinia pseudotuberculosis* infection. Cell Microbiol. 2006;8(9):1504–15. doi: 10.1111/j.1462-5822.2006.00729.x 16922868

[172] Palace S, Proulx M, Szabady R, et al. Gain-of-function analysis reveals important virulence roles for the *Yersinia pestis* type III secretion system effectors YopJ, YopT, and YpkA. Infect Immun. 2018;86(9):e00318–18. doi: 10.1128/IAI.00318-18 29891548 PMC6105907

[173] Galyov E, Hakansson S, Wolf-Watz H. Characterization of the operon encoding the YpkA Ser/Thr protein kinase and the YopJ protein of *Yersinia pseudotuberculosis*. J Bacteriol. 1994;176(15):4543–8. doi: 10.1128/jb.176.15.4543-4548.1994 8045884 PMC196273

[174] Juris S, Rudolph A, Huddler D, et al. A distinctive role for the *Yersinia* protein kinase: actin binding, kinase activation, and cytoskeleton disruption. Proc Natl Acad Sci U S A. 2000;97(17):9431–6. doi: 10.1073/pnas.170281997 10920208 PMC16881

[175] Barz C, Abahji T, Trulzsch K, et al. The *Yersinia* Ser/Thr protein kinase YpkA/YopO directly interacts with the small GTPases RhoA and rac-1. FEBS Lett. 2000;482(1–2):139–43. doi: 10.1016/S0014-5793(00)02045-7 11018537

[176] Wiley D, Nordfeldth R, Rosenzweig J, et al. The Ser/Thr kinase activity of the *Yersinia* protein kinase a (YpkA) is necessary for full virulence in the mouse, mollifying phagocytes, and disrupting the eukaryotic cytoskeleton. Microb Pathog. 2006;40(5):234–43. doi: 10.1016/j.micpath.2006.02.001 16626927

[177] Navarro L, Koller A, Nordfelth R, et al. Identification of a molecular target for the *Yersinia* protein kinase A. Mol Cell. 2007;26(4):465–77. doi: 10.1016/j.molcel.2007.04.025 17531806

[178] Offermanns S, Toombs C, Hu Y, et al. Defective platelet activation in Gαq-deficient mice. Nature. 1997;389(6647):183–186. doi: 10.1038/38284 9296496

[179] LaRock C, Cookson B. The *Yersinia* virulence effector YopM binds caspase-1 to arrest inflammasome assembly and processing. Cell Host Microbe. 2012;12(6):799–805. doi: 10.1016/j.chom.2012.10.020 23245324 PMC3703949

[180] Ratner D, Orning M, Starheim K, et al. Manipulation of interleukin-1β and interleukin-18 production by *Yersinia pestis* effectors YopJ and YopM and redundant impact on virulence. Journal Of Biological Chemistry. 2016;291(19):9894–9905. doi: 10.1074/jbc.M115.697698 26884330 PMC4858993

[181] Schoberle T, Chung L, McPhee J, et al. Uncovering an important role for YopJ in the inhibition of caspase-1 in activated macrophages and promoting *Yersinia pseudotuberculosis* virulence. Infect Immun. 2016;84(4):1062–72. doi: 10.1128/IAI.00843-15 26810037 PMC4807483

[182] Hentschke M, Berneking L, Belmar Campos C, et al. *Yersinia* virulence factor YopM induces sustained RSK activation by interfering with dephosphorylation. PloS One. 2010;5(10):e13165. doi: 10.1371/journal.pone.0013165 20957203 PMC2950144

[183] McDonald C, Vacratsis P, Bliska J, et al. The *Yersinia* virulence factor YopM forms a novel protein complex with two cellular kinases. J Biol Chem. 2003;278(20):18514–23. doi: 10.1074/jbc.M301226200 12626518

[184] Malik H, Bliska J. The pyrin inflammasome and the *Yersinia* effector interaction. Immunol Rev. 2020;297(1):96–107. doi: 10.1111/imr.12907 32721043 PMC7989412

[185] McPhee J, Mena P, Zhang Y, et al. Interleukin-10 induction is an important virulence function of the *Yersinia pseudotuberculosis* type III effector YopM. Infect Immun. 2012;80(7):2519–27. doi: 10.1128/IAI.06364-11 22547545 PMC3416464

[186] Berneking L, Schnapp M, Rumm A, et al. Immunosuppressive *Yersinia* effector YopM binds DEAD box helicase DDX3 to control ribosomal S6 kinase in the nucleus of host cells. PLOS Pathog. 2016;12(6):e1005660. doi: 10.1371/journal.ppat.1005660 27300509 PMC4907486

[187] Ye Z, Uittenbogaard A, Cohen D, et al. Distinct CCR2+Gr1+cells control growth of the Yersinia pestis Δ*yopM* mutant in liver and spleen during systemic plague. Infect Immun. 2011;79(2):674–687. doi: 10.1128/IAI.00808-10 21149593 PMC3028864

[188] Ye Z, Kerschen E, Cohen D, et al. Gr1+ cells control growth of YopM-negative *Yersinia pestis* during systemic plague. Infect Immun. 2009;77(9):3791–806. doi: 10.1128/IAI.00284-09 19581396 PMC2738001

[189] McCoy M, Marre M, Lesser C, et al. The C-terminal tail of *Yersinia pseudotuberculosis* YopM is critical for interacting with RSK1 and for virulence. Infect Immun. 2010;78(6):2584–98. doi: 10.1128/IAI.00141-10 20368345 PMC2876544

[190] Holmstrom A, Rosqvist R, Wolf-Watz H, et al. Virulence plasmid-encoded YopK is essential for *Yersinia pseudotuberculosis* to cause systemic infection in mice. Infect Immun. 1995;63(6):2269–76. doi: 10.1128/iai.63.6.2269-2276.1995 7768608 PMC173296

[191] Zauberman A, Velan B, Mamroud E, et al. Disparity between *Yersinia pestis* and *Yersinia enterocolitica* O: 8 in YopJ/YopP-dependent functions. Adv Exp Med Biol. 2007;603:312–320. 17966427 10.1007/978-0-387-72124-8_28

[192] Peters K, Dhariwala M, Hughes-Hanks J, et al. Early apoptosis of macrophages modulated by injection of *Yersinia pestis* YopK promotes progression of primary pneumonic plague. PloS Path. 2013;9(4):e1003324. doi: 10.1371/journal.ppat.1003324 PMC3636031 23633954

[193] Dewoody R, Merritt P, Houppert A, et al. YopK regulates the *Yersinia pestis* type III secretion system from within host cells. Mol Microbiol. 2011;79(6):1445–61. doi: 10.1111/j.1365-2958.2011.07534.x 21205017 PMC3210821

[194] Holmstrom A, Pettersson J, Rosqvist R, et al. YopK of *Yersinia pseudotuberculosis* controls translocation of Yop effectors across the eukaryotic cell membrane. Molecular Microbiology. 1997;24(1):73–91. doi: 10.1046/j.1365-2958.1997.3211681.x 9140967

[195] Thorslund S, Edgren T, Pettersson J, et al. The RACK1 signaling scaffold protein selectively interacts with *Yersinia pseudotuberculosis* virulence function. PloS One. 2011;6(2):e16784. doi: 10.1371/journal.pone.0016784 21347310 PMC3037380

[196] Aili M, Isaksson E, Carlsson S, et al. Regulation of *Yersinia* yop-effector delivery by translocated YopE. Int J Med Microbiol. 2008;298(3–4):183–92. doi: 10.1016/j.ijmm.2007.04.007 17597003

[197] Ruckdeschel K, Pfaffinger G, Trulzsch K, et al. The proteasome pathway destabilizes *Yersinia* outer protein E and represses its antihost cell activities. The Journal Of Immunology. 2006;176(10):6093–6102. doi: 10.4049/jimmunol.176.10.6093 16670318

[198] Gaus K, Hentschke M, Czymmeck N, et al. Destabilization of YopE by the ubiquitin-proteasome pathway fine-tunes Yop delivery into host cells and facilitates systemic spread of *Yersinia enterocolitica* in host lymphoid tissue. Infect Immun. 2011;79(3):1166–1175. doi: 10.1128/IAI.00694-10 21149597 PMC3067501

[199] Brodsky I, Medzhitov R, Isberg RR. Reduced secretion of YopJ by *Yersinia* limits in vivo cell death but enhances bacterial virulence. PLOS Pathog. 2008;4(5):1–14. doi: 10.1371/journal.ppat.1000067 PMC2361194 18483548

[200] Zauberman A, Tidhar A, Levy Y, et al. *Yersinia pestis* endowed with increased cytotoxicity is avirulent in a bubonic plague model and induces rapid protection against pneumonic plague. PloS One. 2009;4(6):e5938. doi: 10.1371/journal.pone.0005938 19529770 PMC2691952

[201] Peterson L, Philip N, Dillon C, et al. Cell-extrinsic TNF collaborates with TRIF signaling to promote *Yersinia*-induced apoptosis. J Immunol. 2016;197(10):4110–7. doi: 10.4049/jimmunol.1601294 27733552 PMC5123756

[202] Mares C, Lugo F, Albataineh M, et al. Heightened virulence of *Yersinia* is associated with decreased function of the YopJ protein. Infect Immun. 2021;89(12). doi: 10.1128/IAI.00430-21 PMC8594599 34543120

[203] Zheng Y, Lilo S, Brodsky I, et al. A *Yersinia* effector with enhanced inhibitory activity on the NF-κB pathway activates the NLRP3/ASC/caspase-1 inflammasome in macrophages. PloS Path. 2011;7(4):e1002026. doi: 10.1371/journal.ppat.1002026 PMC3080847 21533069

[204] Lemaitre N, Sebbane F, Long D, et al. *Yersinia pestis* YopJ suppresses tumor necrosis factor alpha induction and contributes to apoptosis of immune cells in the lymph node but is not required for virulence in a rat model of bubonic plague. Infect Immun. 2006;74(9):5126–5131. doi: 10.1128/IAI.00219-06 16926404 PMC1594864

[205] Monack D, Mecsas J, Bouley D, et al. *Yersinia*-induced apoptosis in vivo aids in the establishment of a systemic infection of mice. J Exp Med. 1998;188(11):2127–37. doi: 10.1084/jem.188.11.2127 9841926 PMC2212385

[206] Huang Y, Xu W, Zhou R. NLRP3 inflammasome activation and cell death. Cell Mol Immunol. 2021;18(9):2114–27. doi: 10.1038/s41423-021-00740-6 34321623 PMC8429580

[207] Nakajima R, Brubaker R. Association between virulence of *Yersinia pestis* and suppression of gamma interferon and tumor necrosis factor alpha. Infect Immun. 1993;61(1):23–31. doi: 10.1128/iai.61.1.23-31.1993 8418045 PMC302683

[208] Bohn E, Sing A, Zumbihl R, et al. IL-18 (IFN-γ-inducing factor) regulates early cytokine production in, and promotes resolution of, bacterial infection in mice. The Journal Of Immunology. 1998;160(1):299–307. doi: 10.4049/jimmunol.160.1.299 9551984

[209] Dave M, Silva J, Elicabe R, et al. *Yersinia enterocolitica* YopH-deficient strain activates neutrophil recruitment to peyer’s patches and promotes clearance of the virulent strain. Infect Immun. 2016;84(11):3172–81. doi: 10.1128/IAI.00568-16 27550935 PMC5067750

[210] Chung L, Park Y, Zheng Y, et al. The *Yersinia* virulence factor YopM hijacks host kinases to inhibit type III effector-triggered activation of the pyrin inflammasome. Cell Host Microbe. 2016;20(3):296–306. doi: 10.1016/j.chom.2016.07.018 27569559 PMC5025386

[211] Zheng Y, Lilo S, Mena P, et al. YopJ-induced caspase-1 activation in *Yersinia*-infected macrophages: Independent of Apoptosis, linked to necrosis, dispensable for innate Host defense. PloS One. 2012;7(4):e36019. doi: 10.1371/journal.pone.0036019 22563435 PMC3338577

[212] Sivaraman V, Pechous R, Stasulli N, et al. *Yersinia pestis* Activates Both IL-1β and IL-1 Receptor Antagonist to Modulate Lung Inflammation during Pneumonic Plague. PLOS Pathog. 2015;11(3):e1004688. doi: 10.1371/journal.ppat.1004688 25781467 PMC4363893

[213] Prior J, Parkhill J, Hitchen P, et al. The failure of different strains of *Yersinia pestis* to produce lipopolysaccharide O-antigen under different growth conditions is due to mutations in the O-antigen gene cluster. FEMS Microbiol Lett. 2001;197(2):229–33. doi: 10.1111/j.1574-6968.2001.tb10608.x 11313139

[214] Lerouge I, Vanderleyden J. O-antigen structural variation: mechanisms and possible roles in animal/plant–microbe interactions. FEMS Microbiol Rev. 2002;26(1):17–47. doi: 10.1111/j.1574-6976.2002.tb00597.x 12007641

[215] Fang X, Kang L, Qiu Y, et al. *Yersinia enterocolitica* in Crohn’s disease. Front Cell Infect Microbiol. 2023;13:1129996. doi: 10.3389/fcimb.2023.1129996 36968108 PMC10031030

[216] Kawahara K, Tsukano H, Watanabe H, et al. Modification of the structure and activity of lipid a in *Yersinia pestis* lipopolysaccharide by growth temperature. Infect Immun. 2002;70(8):4092–8. doi: 10.1128/IAI.70.8.4092-4098.2002 12117916 PMC128165

[217] Knirel Y, Anisimov A, Kislichkina A, et al. Lipopolysaccharide of the *Yersinia pseudotuberculosis* complex. Biomolecules. 2021;11(10):11. doi: 10.3390/biom11101410 PMC8533242 34680043

[218] Montminy S, Khan N, McGrath S, et al. Virulence factors of *Yersinia pestis* are overcome by a strong lipopolysaccharide response. Nat Immunol. 2006;7(10):1066–73. doi: 10.1038/ni1386 16980981

[219] Rebeil R, Ernst R, Gowen B, et al. Variation in lipid a structure in the pathogenic *Yersiniae*. Mol Microbiol. 2004;52(5):1363–73. doi: 10.1111/j.1365-2958.2004.04059.x 15165239

[220] Rebeil R, Ernst R, Jarrett C, et al. Characterization of late acyltransferase genes of *Yersinia pestis* and their role in temperature-dependent lipid a variation. J Bacteriol. 2006;188(4):1381–8. doi: 10.1128/JB.188.4.1381-1388.2006 16452420 PMC1367257

[221] Reines M, Llobet E, Dahlstrom K, et al. Deciphering the acylation pattern of *Yersinia enterocolitica* lipid a. PloS Path. 2012;8(10):e1002978. doi: 10.1371/journal.ppat.1002978 PMC3486919 23133372

[222] Chandler C, Harberts E, Pelletier M, et al. Early evolutionary loss of the lipid a modifying enzyme PagP resulting in innate immune evasion in *Yersinia pestis*. Proc Natl Acad Sci USA. 2021;117(37):22984–22991. doi: 10.1073/pnas.1917504117 PMC7502761 32868431

[223] Reines M, Llobet E, Llompart C, et al. Molecular basis of *Yersinia enterocolitica* temperature-dependent resistance to antimicrobial peptides. J Bacteriol. 2012;194(12):3173–88. doi: 10.1128/JB.00308-12 22505678 PMC3370846

[224] Valtuena A, Neumann G, Spyrou M, et al. Stone age *Yersinia pestis* genomes shed light on the early evolution, diversity, and eology of plague. Proc Natl Acad Sci. 2022;119:e2116722119. doi: 10.1073/pnas.2116722119 35412864 PMC9169917

[225] Minnich S, Rohde H. A rationale for repression and/or loss of motility by pathogenic *Yersinia* in the mammalian host. Adv Exp Med Biol. 2007;603:298–310. 17966426 10.1007/978-0-387-72124-8_27

[226] Kapatral V, Olson J, Pepe J, et al. Temperature-dependent regulation of *Yersinia enterocolitica* class III flagellar genes. Mol Microbiol. 1996;19(5):1061–71. doi: 10.1046/j.1365-2958.1996.452978.x 8830263

[227] Price P, Jin J, Goldman W. Pulmonary infection by *Yersinia pestis* rapidly establishes a permissive environment for microbial proliferation. Proc Natl Acad Sci, USA. 2012;109(8):3083–8. doi: 10.1073/pnas.1112729109 22308352 PMC3286930

[228] Pechous R, Sivaraman V, Stasulli N, et al. Pneumonic plague: the darker side of *Yersinia pestis*. Trends Microbiol. 2016;24(3):190–7. doi: 10.1016/j.tim.2015.11.008 26698952

[229] Olson R, Dhariwala M, Mitchell W, et al. Modification of the pulmonary MyD88 inflammatory response underlies the role of the *Yersinia pestis* pigmentation locus in primary pneumonic plague. Infect Immun. 2021;89(3):e00595–20. doi: 10.1128/IAI.00595-20 33257532 PMC8097263

[230] Lee-Lewis H, Anderson D. Absence of inflammation and pneumonia during infection with nonpigmented *Yersinia pestis* reveals a new role for the pgm locus in pathogenesis. Infect Immun. 2010;78(1):220–230. doi: 10.1128/IAI.00559-09 19841077 PMC2798233

[231] Kawai T, Akira S. Pathogen recognition with toll-like receptors. Curr Op Immunol. 2005;17(4):338–344. doi: 10.1016/j.coi.2005.02.007 15950447

[232] Ruckdeschel K, Pfaffinger G, Haase R, et al. Signaling of Apoptosis through TLRs critically involves toll/IL-1 receptor domain-containing adapter inducing IFN-β, but not MyD88, in bacteria-infected murine macrophages. The Journal Of Immunology. 2004;173(5):3320–3328. doi: 10.4049/jimmunol.173.5.3320 15322195

[233] Sotolongo J, Espana C, Echeverry A, et al. Host innate recognition of an intestinal bacterial pathogen induces TRIF-dependent protective immunity. J Exp Med. 2011;208(13):2705–16. doi: 10.1084/jem.20110547 22124111 PMC3244044

[234] Rosadini C, Zanoni I, Odendall C, et al. A single bacterial immune evasion strategy dismantles both MyD88 and TRIF signaling pathways downstream of TLR4. Cell Host Microbe. 2015;18(6):682–93. doi: 10.1016/j.chom.2015.11.006 26651944 PMC4685476

[235] Diebold S, Kaisho T, Hemmi H, et al. Innate antiviral responses by means of TLR7-mediated recognition of single-stranded RNA. Science. 2004;303:1529–1531. doi: 10.1126/science.1093616 14976261

[236] Dhariwala M, Olson R, Anderson D, et al. Induction of type I interferon through a noncanonical Toll-like receptor 7 pathway during *Yersinia pestis* infection. Infect Immun. 2017;85(11):e00570–17. doi: 10.1128/IAI.00570-17 28847850 PMC5649010

[237] Patel A, Lee-Lewis H, Hughes-Hanks J, et al. Opposing roles for interferon regulatory factor-3 (IRF-3) and type I interferon signaling during plague. PLOS Pathog. 2012;8(7):e1002817. doi: 10.1371/journal.ppat.1002817 22911267 PMC3406097

[238] Ke Y, Chen Z, Yang R. *Yersinia pestis*: mechanisms of entry into and resistance to the host cell. Front Cell Infect Microbiol. 2013;3:106. doi: 10.3389/fcimb.2013.00106 24400226 PMC3871965

[239] Tsukano H, Kura F, Inoue S, et al. *Yersinia pseudotuberculosis* blocks the phagosomal acidification of B10.A mouse macrophages through the inhibition of vacuolar H+-ATPase activity. Microbial Pathogenesis. 1999;27(4):253–263. doi: 10.1006/mpat.1999.0303 10502466

[240] Pujol C, Bliska J. The ability to replicate in macrophages is conserved between *Yersinia pestis* and *Yersinia pseudotuberculosis*. Infect Immun. 2003;71(10):5892–9. doi: 10.1128/IAI.71.10.5892-5899.2003 14500510 PMC201058

[241] Grabenstein J, Marceau M, Pujol C, et al. The response regulator PhoP of *Yersinia pseudotuberculosis* is important for replication in macrophages and for virulence. Infect Immun. 2004;72(9):4973–4984. doi: 10.1128/IAI.72.9.4973-4984.2004 15321989 PMC517447

[242] Vadyvaloo V, Viall A, Jarrett C, et al. Role of the PhoP–PhoQ gene regulatory system in adaptation of *Yersinia pestis* to environmental stress in the flea digestive tract. Microbiology (Reading). 2015;161(6):1198–1210. doi: 10.1099/mic.0.000082 25804213 PMC4635514

[243] Pujol C, Klein K, Romanov G, et al. *Yersinia pestis* can reside in autophagosomes and avoid xenophagy in murine macrophages by preventing vacuole acidification. Infect Immun. 2009;77(6):2251–61. doi: 10.1128/IAI.00068-09 19289509 PMC2687347

[244] Connor M, Pulsifer A, Chung D, et al. *Yersinia pestis* targets the host endosome recycling pathway during the biogenesis of the *Yersinia*-containing vacuole to avoid killing by macrophages. MBio. 2018;9(1). doi: 10.1128/mBio.01800-17 PMC5821078 29463656

[245] Pujol C, Grabenstein J, Perry R, et al. Replication of *Yersinia pestis* in interferon γ-activated macrophages requires ripA , a gene encoded in the pigmentation locus. Proc Natl Acad Sci USA. 2005;102(36):12909–12914. doi: 10.1073/pnas.0502849102 16120681 PMC1200267

[246] Pujol C, Bliska J. Turning *Yersinia* pathogenesis outside in: subversion of macrophage function by intracellular yersiniae. Clin Immunol. 2005;114(3):216–26. doi: 10.1016/j.clim.2004.07.013 15721832

[247] Bozue J, Mou S, Moody K, et al. The role of the *phoPQ* operon in the pathogenesis of the fully virulent CO92 strain of *Yersinia pestis* and the IP32953 strain of *Yersinia pseudotuberculosis*. Microb Pathog. 2011;50(6):314–21. doi: 10.1016/j.micpath.2011.02.005 21320584

[248] Fisher M, Castillo C, Mecsas J. Intranasal inoculation of mice with *Yersinia pseudotuberculosis* causes a lethal lung infection that is dependent on Yersinia outer proteins andPhoP. Infect Immun. 2007;75(1):429–442. doi: 10.1128/IAI.01287-06 17074849 PMC1828392

[249] Virgin H, Levine B. Autophagy genes and immunity. Nat Immunol. 2009;10(5):461–570. doi: 10.1038/ni.1726 19381141 PMC2715365

[250] Eisele N, Brown C, Anderson D. Phagocytes and humoral immunity to pneumonic plague. Adv Exp Med Biol. 2012;954:165–171. 22782760 10.1007/978-1-4614-3561-7_21

[251] Moreau K, Lacas-Gervais S, Fujita N, et al. Autophagosomes can support *Yersinia pseudotuberculosis* replication in macrophages. Cell Microbiol. 2010;12(8):1108–23. doi: 10.1111/j.1462-5822.2010.01456.x 20180800

[252] Lopez M, Schimmeck H, Gopengieber J, et al. Activation of the macroautophagy pathway by *Yersinia enterocolitica* promotes intracellular multiplication and egress of yersiniae from epithelial cells. Cellular Microbiology. 2019;21(9):e13046. doi: 10.1111/cmi.13046 31099152

